# BTG2 bridges PABPC1 RNA-binding domains and CAF1 deadenylase to control cell proliferation

**DOI:** 10.1038/ncomms10811

**Published:** 2016-02-25

**Authors:** Benjamin Stupfler, Catherine Birck, Bertrand Séraphin, Fabienne Mauxion

**Affiliations:** 1 Institut de Génétique et de Biologie Moléculaire et Cellulaire, 67404 Illkirch, France; 2 Centre National de la Recherche Scientifique UMR7104, 67404 Illkirch, France; 3 Institut National de la Santé et de la Recherche Médicale U964, 67404 Illkirch, France; 4 Université de Strasbourg, 67404 Illkirch, France

## Abstract

While BTG2 plays an important role in cellular differentiation and cancer, its precise molecular function remains unclear. BTG2 interacts with CAF1 deadenylase through its APRO domain, a defining feature of BTG/Tob factors. Our previous experiments revealed that expression of BTG2 promoted mRNA poly(A) tail shortening through an undefined mechanism. Here we report that the APRO domain of BTG2 interacts directly with the first RRM domain of the poly(A)-binding protein PABPC1. Moreover, PABPC1 RRM and BTG2 APRO domains are sufficient to stimulate CAF1 deadenylase activity *in vitro* in the absence of other CCR4–NOT complex subunits. Our results unravel thus the mechanism by which BTG2 stimulates mRNA deadenylation, demonstrating its direct role in poly(A) tail length control. Importantly, we also show that the interaction of BTG2 with the first RRM domain of PABPC1 is required for BTG2 to control cell proliferation.

## Abstract (web summary)

BTG2 promotes mRNA poly(A) tail shortening and regulates cellular differentiation. Here, Stupfler *et al*. show that the BTG2 APRO domain interacts with PABPC1 RRM1, allowing the former to recruit and stimulate the poly(A) tail shortening activity of CAF1 deadenylase and to control cell proliferation.

## Graphical abstract

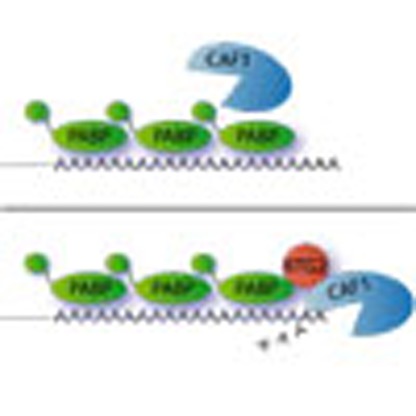

The BTG2 factor, also known as TIS21 or PC3, is a member of a family of proteins found in metazoans, the BTG/Tob family. BTG/Tob factors are characterized by the presence of a conserved domain at their N terminus, the APRO (AntiPROliferative) or BTG domain^1^. In mammals, six family members have been identified. Among the BTG factors, BTG1 and BTG2 are highly similar proteins, while BTG3 and BTG4 are more distantly related. The two Tob proteins are also very similar to each other and depart from BTG factors by containing PAM2 (Poly(A)-binding-protein-interacting Motif 2) peptides in their C-terminal tails^2^. These peptides mediate the interaction of Tob proteins with the C-terminal MLLE domain of cytoplasmic Poly(A)-binding proteins (PABPC)^3^,^4^,^5^. Importantly, BTG/Tob proteins display antiproliferative properties as their ectopic expression in a variety of cell lines reduced cell proliferation (for a recent review, see ref. 6).

Interest for BTG2 was stimulated by its implication in cellular differentiation, in particular during neuronal development. BTG2 expression is precisely regulated spatiotemporally during embryonic and adult neurogenesis^7^,^8^,^9^. Its overexpression during neurogenesis led to microcephaly^10^,^11^, while its deletion triggered impaired neuronal differentiation^12^. BTG2 knockout mice showed also abnormalities in vertebral patterning^13^, supporting a general role in embryonic development and cell differentiation. Furthermore, BTG2 is a direct transcriptional target of p53 (ref. 14) and has been shown to be a major effector of suppression of Ras-induced transformation by p53 (ref. 15). Consistently, BTG2 expression is frequently reduced in human cancers^16^,^17^ and its expression correlates with tumour grade and prognosis^18^,^19^. However, despite its importance in several biological processes and in human cancers, the precise molecular function of BTG2 is not well defined.

BTG2, as well as all the BTG/Tob proteins that have been tested, interacts directly with CAF1, a subunit of the CCR4–NOT complex^20^. The latter complex contains two interacting deadenylases, CAF1 and CCR4 (ref. 21), that have been shown to catalyse the major phase of the poly(A)-tail-shortening initiating mRNA decay^22^,^23^,^24^. In vertebrates, two CAF1 paralogues, CNOT7 and CNOT8 (that we refer here collectively as CAF1 deadenylase), and two CCR4 paralogues, CNOT6 and CNOT6L, coexist^25^. Deadenylation has been shown to be precisely controlled, thus contributing to the regulation of gene expression. Consistently, recruitment of the CCR4–NOT complex to the 3′ untranslated region of specific subsets of mRNAs by various mRNA decay regulators, including microRNA-associated factors, is a prevailing mechanism to destabilize mRNA and thus to modulate gene expression^26^,^27^,^28^,^29^.

Earlier results suggested that BTG2 could function as transcription cofactor^30^,^31^,^32^, but the interaction of BTG/Tob factors with CAF1 deadenylase indicated that BTG/Tob proteins could also be implicated in poly(A)-tail length control. Consistently, ectopic expression of Tob and BTG2 proteins stimulated deadenylation of reporter and endogenous transcripts^5^,^33^. On the basis of the capacity of Tob proteins to bind simultaneously CAF1 and the MLLE domain of PABPC, a mechanism for deadenylation activation by Tob proteins was proposed: Tob factors would recruit the CCR4–NOT complex to PABPC that is bound to the mRNA poly(A) tail. This hypothesis was confirmed by showing that tethering directly Tob to mRNA abolished the requirement for PABPC binding^34^. The absence of PAM2 motif in BTG2 suggested that an alternative mechanism might be involved in BTG2 stimulation of mRNA deadenylation.

Deciphering this mechanism, we discovered that the BTG2 and BTG1 APRO domains, but not the Tob1 APRO domain, bind directly to the first RRM domain of PABPC1. We show further that this interaction is sufficient *in vitro* and necessary *in cellulo* for BTG2 to stimulate mRNA deadenylation. Moreover, the interaction between BTG2 and PABPC1 is required for BTG2 to exert its antiproliferative function.

## Results

### BTG2 APRO domain is sufficient to stimulate deadenylation

**Figure 1:**
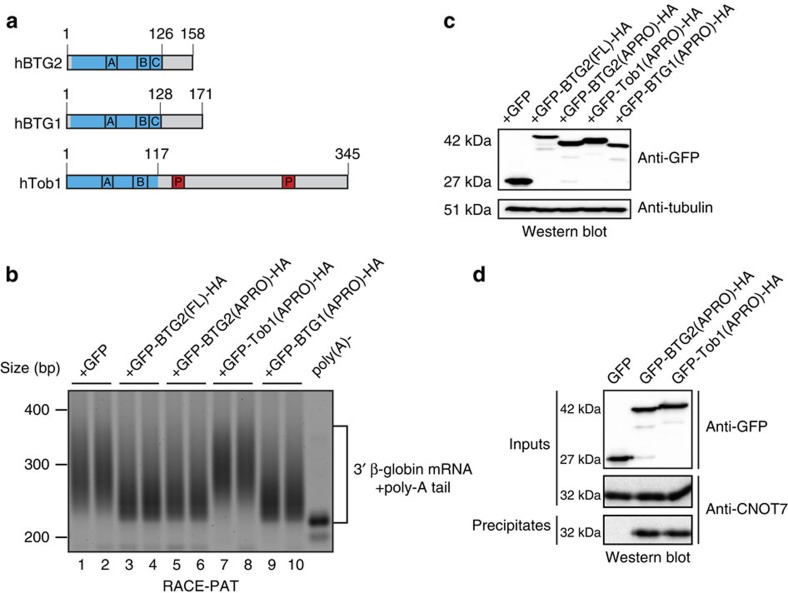
APRO domains of BTG2 and BTG1 but not of Tob1 are sufficient to stimulate deadenylation of a reporter transcript. (**a**) Schematic organization of human BTG2, BTG1 and Tob1 proteins. APRO domains are highlighted in blue. The conserved boxA (A) and boxB (B) motifs (signatures for the BTG/Tob family), the boxC motif (C; specific for BTG1 and BTG2, see Supplementary Fig. 7) and the PAM2 motifs (P; found in the C-terminal region of Tob1) are indicated. Sequence identity/similarity between hBTG2 and hBTG1 APRO domains are 68.0%/79.7%, respectively, while sequence identity/similarity between hBTG2 and hTob1 APRO domains are 38.6%/58.3% respectively. (**b**) Reverse transcription polymerase chain reaction amplification of the poly(A) tails of the β-globin reporter transcript. HEK293 Tet-Off cells were co-transfected with plasmids expressing the β-globin reporter and the different HA-tagged BTG/Tob GFP fusion proteins (or empty GFP expression vector). Fragments comprising ∼200 bp of the 3′ end of the β-globin mRNA in addition to poly(A) tails were amplified using a RACE-PAT assay. An RNA sample treated with oligo(dT) and RNase H was used as a control for deadenylated β-globin mRNA (poly(A)-). Experiment was repeated twice with two biological duplicates. Quantification of the gel gave an estimated average poly(A) tail length (mean±s.d., *n*=2) of 74.8±1.6, 43.3±1.1, 46.8±3.7, 79±3.4 and 37.4±0.2 nucleotides in cells expressing GFP, GFP-BTG2(FL)-HA, GFP-BTG2(APRO)-HA, GFP-Tob1(APRO)-HA and GFP-BTG1(APRO)-HA, respectively. (**c**) A western blot analysis of the cell lysates that corresponded to the transfections performed in **b** is shown to demonstrate expression of the GFP fusion proteins. (**d**) Co-immunoprecipitation of CAF1 (CNOT7 paralogue) with BTG2 and Tob1 APRO domains. HEK293 cells were transfected with plasmids expressing HA-tagged BTG/Tob GFP fusion proteins or empty GFP expression vector. Proteins were precipitated with anti-HA agarose beads and revealed by western blot with anti-GFP and anti-CNOT7 antibodies.

Unlike Tob factors, the short BTG2 C-terminal region following the APRO domain does not contain PAM2 motifs but has been shown to be the target of phosphorylation and ubiquitination^35^,^36^. To test whether the C-terminal tail of BTG2 is necessary for stimulation of mRNA deadenylation, as it is the case for Tob, haemagglutinin (HA)-tagged full-length (FL) and progressively truncated BTG2 proteins were expressed in HEK293 Tet-Off cells together with a β-globin mRNA reporter expressed under the control of a tetracycline-regulated promoter. Transcriptional pulse-chase experiments revealed that the BTG2 protein in which the C-terminal region has been totally deleted was as efficient to induce deadenylation as the FL protein (Supplementary Fig. 1). This result was confirmed with a RACE-PAT assay in which the poly(A) tail length of the β-globin transcript is visualized using reverse transcription polymerase chain reaction (PCR) reactions. In this assay, the reporter co-expressed with a GFP (green fluorescent protein)-BTG2(APRO)-HA fusion protein (amino acids 1–126 of BTG2; Fig. 1a) presented at steady-state shorter poly(A) tails than the reporter transfected with empty expression plasmid, similarly as it was observed in the cells co-expressing the FL BTG2 protein (Fig. 1b, compare lanes 3–4 and 5–6 to lanes 1–2). Thus, the APRO domain of BTG2 is sufficient to stimulate mRNA deadenylation.

To test whether this feature is a conserved property of the APRO domains of the BTG/Tob proteins, BTG1 or Tob1 APRO domains (amino acids 1–128 or 1–117, respectively, Fig. 1a) were also expressed in HEK293 Tet-Off cells with the β-globin reporter. N-terminal fusion of GFP to the APRO domains allowed comparable expression levels (Fig. 1c). RACE-PAT assays revealed that in cells expressing GFP-BTG1(APRO)-HA, like in the cells expressing GFP-BTG2(FL)-HA or GFP-BTG2(APRO)-HA, the poly(A) tails of the β-globin transcript were shorter than in control cells (Fig. 1b, compare lanes 9–10 to lanes 1–2). By contrast, this was not the case in the presence of GFP-Tob1(APRO)-HA (Fig. 1c, lanes 7–8). The latter observation is in agreement with previous reports that showed that the PAM2 motifs present in the C-terminal region of Tob1 are necessary for Tob1 to induce mRNA deadenylation^4^,^5^. Nevertheless, to ascertain that this result is not due to the inability of GFP-Tob1(APRO)-HA to interact with CAF1 deadenylase, co-precipitation experiments were performed. Western blot analysis confirmed that GFP-Tob1(APRO)-HA, like GFP-BTG2(APRO)-HA, co-precipitated CNOT7, one of the two CAF1 paralogues found in humans (Fig. 1d). Altogether, these results demonstrate that the APRO domains of the two closely related BTG1 and BTG2 proteins are sufficient to stimulate mRNA deadenylation, whereas the more distantly related Tob1 APRO domain is not. This result suggested that specific partners of the BTG1/BTG2 APRO domains could be involved in BTG-activated deadenylation.

### BTG2 APRO interacts with PABPC1 RRM1

**Figure 2:**
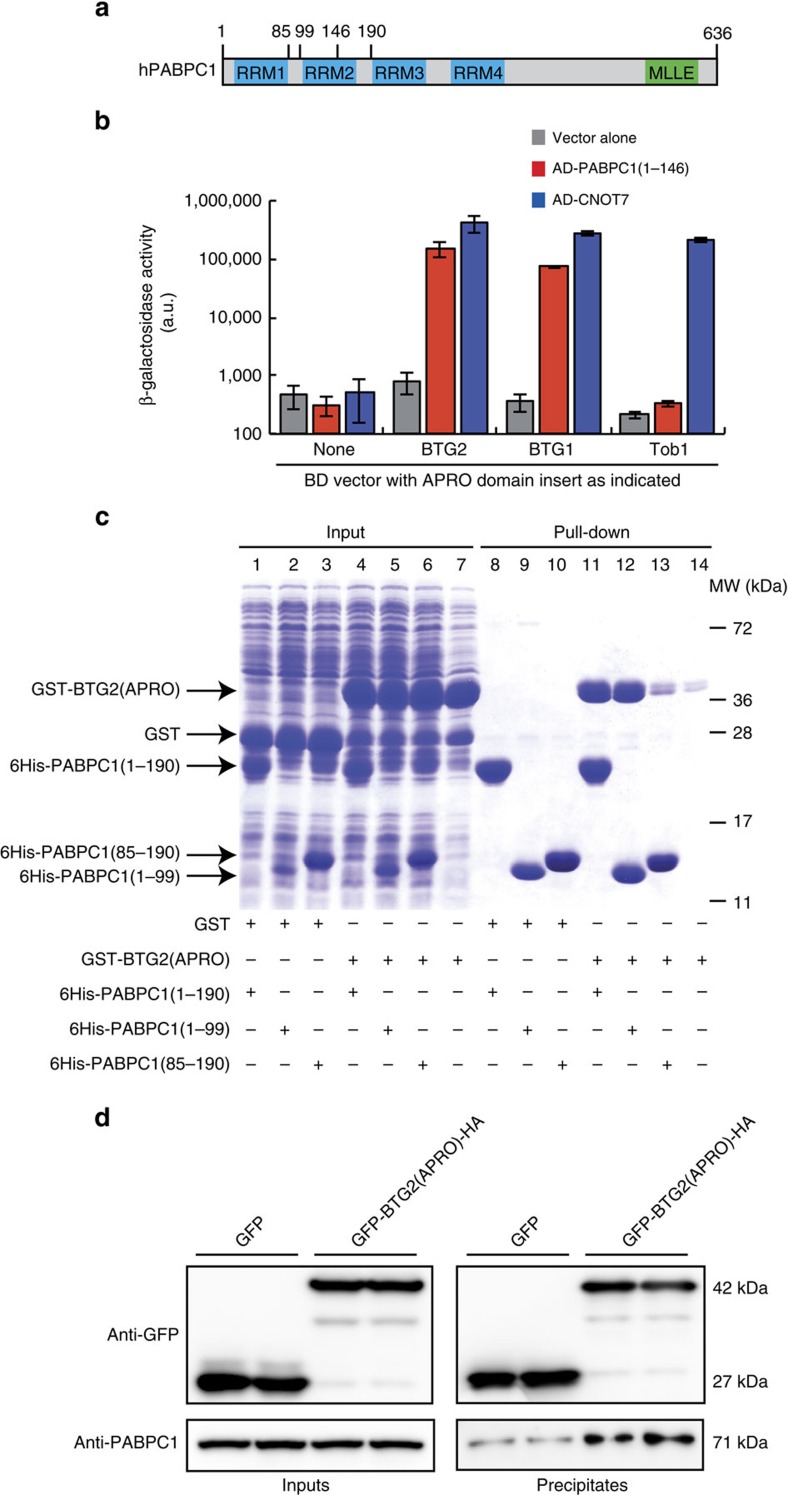
The APRO domain of BTG2 interacts directly with the first RRM domain of PABPC1. (**a**) Organization of the human PABPC1 protein. The RRM and C-terminal MLLE domains are highlighted. Amino-acid positions relevant for this study are shown. (**b**) Interaction of BTG2, BTG1 and Tob1 APRO domains with CAF1 (CNOT7 paralogue) and the first RRM domains of PABPC1 in yeast two-hybrid assay. Interaction between the different chimeric proteins indicated was monitored by β-galactosidase assays. Activities are expressed in arbitrary units (a.u.). Experiment was performed twice with two biological duplicates. Error bars (s.d.) correspond to two biological replicates. (**c**) Co-purification experiments showing direct interaction between BTG2(APRO) and the first RRM domain of PABPC1. GST- and His-recombinant proteins were co-expressed in *E. coli* as indicated. His-tagged proteins were purified with Nickel beads. Proteins were resolved on 15% SDS–PAGE gels and stained with Coomassie blue. (**d**) Co-immunoprecipitation of PABPC1 with BTG2 APRO domain. HEK293 cells were transfected with plasmids expressing GFP-BTG2(APRO)-HA or GFP alone. Twenty-four hours after transfection, cells were treated with Dithio-bis Succinimidyl Propionate 0.25 mM and lysed in the presence of RNAse A. Proteins were then precipitated with GFP-trap beads and revealed by western blot with anti-GFP and anti-PABPC1 antibodies. Three biological replicates were performed, two being shown.

To identify interacting partners of BTG2 APRO domain, a large-scale yeast two-hybrid screen was conducted with a LexA Binding Domain fused to BTG2 APRO as bait. One of the positive clones encoded part of the first two RRM domains of PABPC1, the major mammalian PABPC (amino acids 1–146; Fig. 2a). Interaction of these domains with the APRO domains of BTG1 and Tob1 was then tested in the two-hybrid system by monitoring β-galactosidase production. This assay revealed an interaction of the first two RRM domains of PABPC1 with the APRO domains of BTG1 and BTG2, but not with the APRO domain of Tob1 (Fig. 2b). The latter did not result from the absence of expression of the Tob1 APRO domain as it interacted with CAF1 in this assay (Fig. 2b, AD-CNOT7). Interestingly, the specific interaction of the BTG1/BTG2 APRO domains with the first two RRMs of PABPC1 correlated with their ability to activate deadenylation (Fig. 1b).

To determine whether the APRO domain of BTG2 and the first RRM domains of PABPC1 are able to interact directly, recombinant proteins consisting of the APRO domain of BTG2 fused to glutathione *S*-transferase (GST-BTG2(APRO)) and 6His-tagged PABPC1 RRM domains 1 and 2 (amino acids 1–190, 6His-PABPC1(1–190)) were expressed in *Escherichia coli*. Pull-down (PD) assays performed with Ni-NTA beads demonstrated that GST-BTG2(APRO) co-purified with 6His-PABPC1(1–190) when both proteins were co-expressed (Fig. 2c, lane 11), whereas the GST protein did not (Fig. 2c, lane 8). As GST-BTG2(APRO) expressed alone showed only background binding to the Ni-NTA beads (Fig. 2c, lane 14), these results indicated that recombinant BTG2 APRO domain binds specifically to the first two RRMs of PABPC1. We further purified the BTG2(APRO) protein, after protease cleavage to remove the GST part, and 6His-PABPC1(1–190) protein to homogeneity and performed analytical ultracentrifugation experiments. This confirmed that BTG2(APRO) and 6His-PABPC1(1–190) can form a 1:1 complex with a *K*_d_ of approximately 5 μM and demonstrated that no RNA is required for the formation of the complex (Supplementary Fig. 2). The first (amino acids 1–99) and second (amino acids 85–190) RRM domains of PABPC1 were also expressed independently as His-tagged recombinant proteins. PD assays showed that GST-BTG2(APRO) co-purified as efficiently with 6His-PABPC1(1–99) as with 6His-PABPC1(1–190) (Fig. 2c, lanes 11 and 12), whereas it was found only in amount slightly above background when it was co-expressed with 6His-PABPC1(85–190) (Fig. 2c, lane 13). We concluded thus that the first RRM domain of PABPC1 is sufficient for the interaction with the BTG2 APRO domain.

To test whether BTG2 binding to PABPC1 is competing with its interaction with CAF1, we performed PD and analytical ultracentrifugation analyses. This revealed that BTG2 interactions with PABPC1 and CAF1 are not mutually exclusive and that formation of a trimeric complex does occur (Supplementary Fig. 3a,b).

GFP and GFP-BTG2(APRO)-HA proteins were then expressed in HEK293 cells in duplicate and immunoprecipitated using the GFP-Trap matrix. In these conditions, GFP-BTG2(APRO)-HA precipitated reproducibly endogenous PABPC1 significantly above the background observed with GFP alone (Fig. 2d), demonstrating that the BTG2/PABPC1 complex forms *in cellulo*.

Altogether, our results establish that BTG2 and BTG1, similarly to Tob1 and Tob2, interact directly with PABPC1. However, the domains involved in the interaction are unexpectedly different, an unusual situation for related proteins: while Tob1 and Tob2 interact with the C-terminal MLLE domain of PABPC1 through PAM2 motifs like many other proteins^37^, BTG2 (and likely BTG1) interaction with PABPC1 involves the APRO domain of the former and the first RRM domain of the latter.

### BTG2 and PABPC1 stimulate CAF1 deadenylase activity *in vitro*

**Figure 3:**
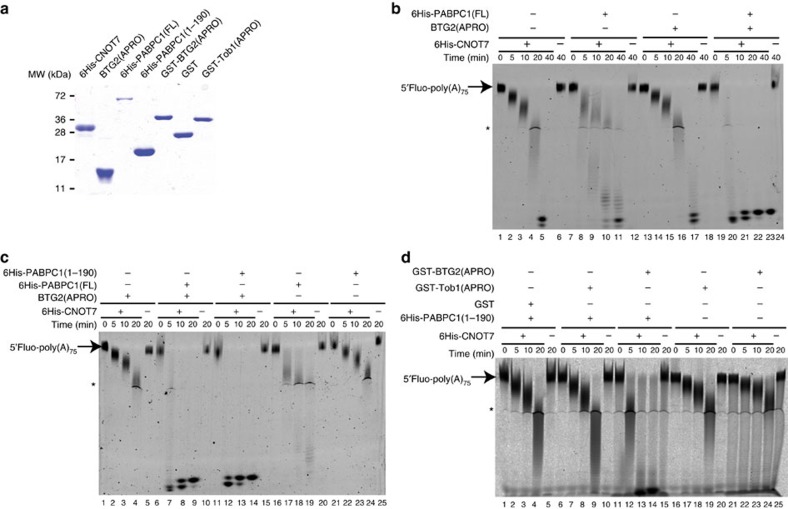
BTG2 APRO domain and PABPC1 stimulates CAF1 (CNOT7 paralogue) deadenylase activity *in vitro.* (**a**) SDS–PAGE analysis of purified recombinant proteins used in *in vitro* deadenylation assays. Five micrograms of purified proteins were resolved on 12% SDS–PAGE gel stained with Coomassie blue. Reduced amount of 6His-PABPC1(FL) likely reflects inaccurate protein concentration determination because of the presence of shorter protein fragments. (**b**) *In vitro* deadenylation assays with CAF1 (CNOT7 paralogue), BTG2(APRO) and full-length PABPC1. A 5′ Fluorescein-labelled poly(A) substrate of approximately 75 residues was incubated in defined conditions (see Methods), with purified 6His-CNOT7, BTG2(APRO) and/or 6His-PABPC1(FL) as indicated. Reaction products were fractionated on 8% denaturing polyacrylamide gel. HEPES present in the buffer induced an altered migration of some products (asterisk). The apparent increased size of the final product observed at the latest time points is an artefact resulting from the increased impact of the hydrophobic fluorophore on the migration of shorter RNA fragments. Experiment was performed three times. Deadenylation rates are: 1.8, 1.5, 1.9 and 14.1 nts per min for 6His-CNOT7, either alone or with 6His-PABPC1(FL), BTG2(APRO) or both, respectively. (**c**) *In vitro* deadenylation assays with CAF1 (CNOT7 paralogue), BTG2(APRO), full-length or first RRM domains of PABPC1. Same as in **b**. Altered migration of the substrate was sometimes observed at early time points, particularly in the presence of 6His-PABPC1 (1–190). This varied between experiments and is partly because of the presence of the PABPC1 proteins as it is not observed after phenol–chloroform extraction of the samples. Experiment was repeated twice. Determined deadenylation rates in nts per min are 2.1 with BTG2(APRO), 13.7 with BTG2(APRO)+6His-PABPC1(FL), 14.3 with BTG2(APRO)+6His-PABPC1(1–190), 2.8 with 6HisPABPC1(FL) and 2.0 with 6HisPABPC1(1–190). (**d**) *In vitro* deadenylation with CAF1 (CNOT7 paralogue), BTG2 or Tob1 APRO domains, and first RRM domains of PABPC1. Same as in **b**. Experiment was performed three times. CAF1 deadenylation rates in nts per min are 2.3 with GST+6HisPABPC1(1–190), 2.7 with GST-Tob1(APRO)+6HisPABPC1(1–190), 7.2 with GST-BTG2(APRO)+6HisPABPC1(1–190), 2.1 with GST-Tob1(APRO) and 1.7 with GST-BTG2(APRO). The stimulation by GST-BTG2(APRO)+6HisPABPC1(1–190) is decreased in this experiment because the GST fusion proteins were batched and not HPLC-purified, and thus contained more contaminants.

We then tested whether the newly identified interaction between BTG2 and PABPC1 could have an impact on CAF1 deadenylase activity by performing *in vitro* deadenylation assays. To this end, BTG2(APRO) as well as 6His-tagged CNOT7 and FL PABPC1 proteins were purified to homogeneity (Fig. 3a). Because PABPC1 binds cooperatively to poly(A)^38^,^39^,^40^ and this could influence deadenylation, a 5′-fluorescein-labelled RNA substrate consisting of approximately 75A residues, that in theory can accommodate the binding of three PABPC1 molecules, was used as substrate. Gel-mobility shift confirmed that multiple PABPC1 molecules bound this substrate and allowed the selection of a PABPC1 concentration ensuring full substrate coverage for downstream assays (Supplementary Fig. 4a,b). During the deadenylation assays, the poly(A) substrate was progressively degraded by 6His-CNOT7 in a time course of 40 min (Fig. 3b, lanes 1–5). Polyacrylamide gel electrophoresis revealed that the size of the reaction end product was less than four residues (Supplementary Fig. 5). Quantification of Fig. 3b indicated a deadenylation rate of 1.8 nucleotides per minute (nts per min) in these conditions. No major difference in RNA degradation rates could be detected after independent addition to the reaction of BTG2(APRO) (Fig. 3b, lanes 13–17, deadenylation rate 1.9 nts per min) or of 6His-PABPC1(FL) (Fig. 3b, lanes 7–11, deadenylation rate 1.5 nts per min). By contrast, a striking acceleration of deadenylation was observed when both BTG2(APRO) and 6His-PABPC1(FL) were added: in their simultaneous presence, the deadenylation rate was at least 14.1 nts per min, thus over seven times faster than with CAF1 alone (Fig. 3b). No degradation of the substrate was detected when it was incubated with 6His-PABPC1(FL), BTG2(APRO) or both in absence of 6His-CNOT7 (Fig. 3b, lanes 12, 18 and 24, respectively). Thus, the simultaneous addition of PABPC1 and of the APRO domain of BTG2 stimulates strongly CAF1 deadenylase activity *in vitro*.

A similar result was observed when only the first two RRM domains of PABPC1 were added in the deadenylation reaction, together with BTG2(APRO) (Fig. 3c, lanes 11–14, deadenylation rate 14.3 nts per min), instead of FL PABPC1 (Fig. 3c, lanes 6–9, deadenylation rate 13.7 nts per min). This demonstrates that oligomerization of PABPC1, which requires the linker and MLLE domains^38^,^39^,^40^, is not involved in the mechanism of stimulation of CAF1 deadenylase activity by BTG2 and PABPC1. Reactions performed with shorter time course or reduced amount of CAF1 revealed that the presence of BTG2 APRO and PABPC RRMs stimulate CAF1 activity without changing its progressive catalytic mode (Supplementary Fig. 6a,b).

To test whether the capacity to stimulate CAF1 deadenylase activity in the presence of PABPC1 is specific for BTG2, the APRO domains of BTG2 and Tob1 were purified as GST fusion proteins (Fig. 3a). In the presence of 6His-PABPC1(1–190), GST-BTG2(APRO) boosted 6His-CNOT7 deadenylase activity, as expected (Fig. 3d, lanes 11–14), whereas addition of GST-Tob1(APRO) did not (Fig. 3d, lanes 6–9). This result shows that the capacity to stimulate CAF1 deadenylase activity *in vitro* is not a general property of the BTG/Tob APRO domains.

### Deadenylation activation requires BTG2–PABPC1 interaction

**Figure 4:**
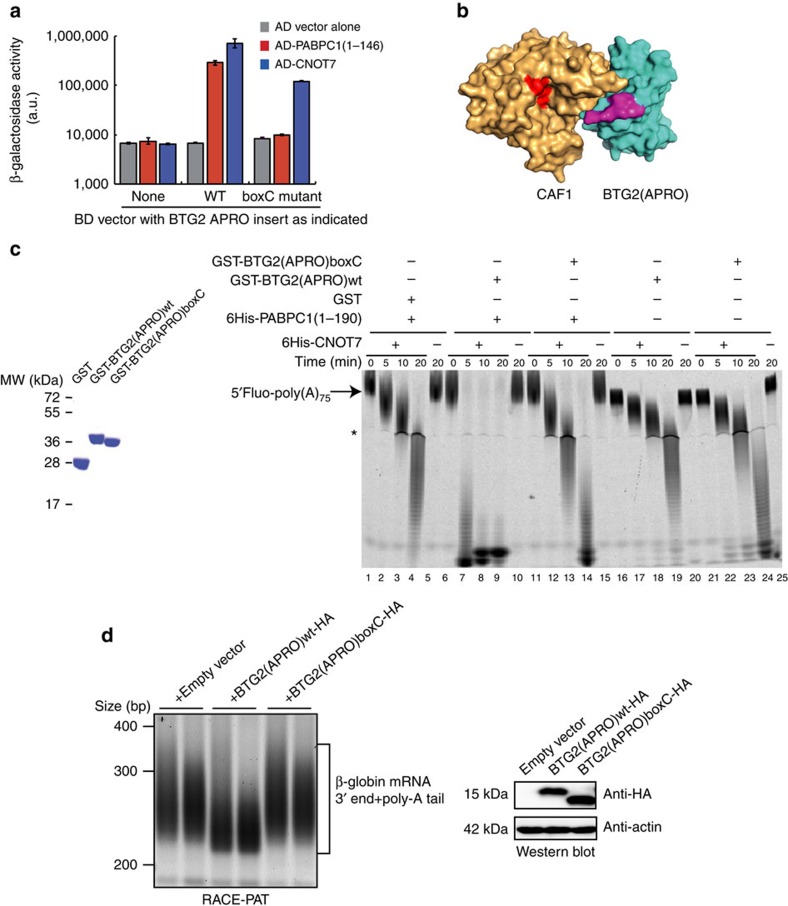
Mutation of the boxC motif impairs BTG2 APRO domain binding to PABPC1 RRM domains and mRNA deadenylation. (**a**) Interaction of wild-type (WT) and mutant BTG2 APRO domains with the first RRM domains of PABPC1 and CAF1 (CNOT7 paralogue) in yeast two-hybrid assay. The sequence DGSIC of boxC BTG2 motif (amino acids 116–120) was substituted by the sequence found in the Tob1 APRO domain (KGPVK), and the interaction of WT (BD-BTG2(APRO)wt) and boxC-mutated (BD-BTG2(APRO)boxC) BTG2 APRO domains with PABPC1 RRMs (AD-PABPC1(1–146)) and CAF1 (AD-CNOT7) was monitored by β-galactosidase assays. Activities are expressed in a.u. Error bars (s.d.) correspond to two biological replicates. (**b**) Model of the CAF1-BTG2(APRO) complex structure. The previously solved structures of CAF1-Tob1(APRO) (PDB ID 2D5R) and BTG2(APRO) (PDB ID 3E9V) were used to construct a model for the CAF1–BTG2(APRO) structure. CAF1 is shown in light orange with the conserved active-site residues (DEDHD) in red. BTG2 is coloured in blue and the boxC motif (DGSIC sequence) is in magenta. (**c**) *In vitro* deadenylation with CAF1 (CNOT7 paralogue), first RRM domains of PABPC1 and BTG2 WT or boxC-mutated APRO domains. Five micrograms of the GST fusion proteins used are shown in right panel; the 6His-CNOT7 and 6His-PABPC1(1–190) proteins used are presented in Fig. 3a. Reaction conditions for the *in vitro* experiments are the same as in Fig. 3b. Experiment was performed twice. CAF1 deadenylation rates in nts per min are 2.7 with GST+6HisPABPC1(1–190), 14.4 with GST-BTG2(APRO)wt+6HisPABPC1(1–190), 3.4 with GST-BTG2(APRO)boxC+6HisPABPC1(1–190), 2.1 with GST-BTG2(APRO)wt and 3.5 with GST-BTG2(APRO)boxC. (**d**) Poly(A)-tail length of the β-globin reporter transcript co-expressed with WT and mutant BTG2 APRO domains. HEK293 Tet-Off cells were co-transfected with plasmids expressing the β-globin reporter and WT or boxC-mutated BTG2 APRO domains or empty expression vector. Fragments comprising approximately 200 bp of the 3′ end of the β-globin mRNA in addition to poly(A) tails were amplified using a RACE-PAT assay. Two biological duplicates are shown. A western blot analysis of corresponding cell lysates is shown to demonstrate expression of the BTG2 proteins.

To identify mutations that would impair PABPC1 interaction without affecting CAF1 binding, we performed site-directed mutagenesis of the BTG2 APRO domain by targeting residues (i) conserved between BTG1 and BTG2 but not in Tob APRO domains and (ii) that are exposed at the surface as determined from the published BTG2 APRO structure^41^. The resulting mutants were tested by production of β-galactosidase in the yeast two-hybrid assay. Among the mutants tested, substitution of conserved residues within the boxC motif prevented interaction with PABPC1 but not association with CAF1 (Fig. 4a). The boxC motif has been characterized by others^42^ as a sequence highly conserved in BTG1 and BTG2 proteins but absent from Tob factors (Supplementary Fig. 7) whose deletion impairs the interaction between BTG1 or BTG2 and PRMT1 (Protein arginine N-methyltransferase 1). The BTG2 boxC mutant that we engineered does not contain a deletion of this element but substitution of residues 116–120 of BTG2 (DGSIC) with the sequence found at this location in Tob1 (KGPVK) (see Supplementary Fig. 7) to ensure proper folding of the APRO domain. PD analysis with recombinant proteins expressed in *E. coli* confirmed that this mutation of boxC in the BTG2 APRO domain impaired BTG2 binding to PABPC1 first RRM domain but not to CAF1 (Supplementary Fig. 8a). The substitution of the boxC sequence did not impair PRMT1 binding to BTG2 (Supplementary Fig. 8b), unlike its deletion^42^. Thus, our mutation specifically affects the interaction of BTG2 with PABPC1. Interestingly, a model of the structure of the BTG2(APRO)–CAF1 complex, based on the previously published BTG2(APRO) and Tob1(APRO)–CAF1 structures^41^,^43^, reveals that the boxC motif is present on a BTG2 surface close to the CAF1 catalytic site (Fig. 4b).

We then tested the activity of this BTG2 mutant in *in vitro* deadenylation assays. This showed that addition of the GST-BTG2(APRO)boxC mutant by itself weakly stimulated CAF1 deadenylase activity (Fig. 4c, lanes 21–24, deadenylation rate 3.5 nts per min compared with 2.7 nts per min with GST alone). Importantly, no further increase in CAF1 activity was observed when PABPC1 RRM domains were also present in the reaction (Fig. 4c, lanes 11–14, deadenylation rate 3.4 nts per min), in contrast to what is observed after simultaneous addition of wild-type BTG2 APRO and PABPC1 RRM domains (Fig. 4c, lanes 6–9, deadenylation rate 14.4 nts per min). Thus, the strong stimulation of CAF1 activity that is observed *in vitro* in the presence of BTG2 APRO and PABPC1 RRM domains requires a direct interaction between BTG2 and PABPC1.

The boxC-mutated BTG2 APRO domain was also co-expressed in HEK293 Tet-Off cells with the β-globin reporter to test its ability to stimulate mRNA deadenylation *in cellulo*. RACE-PAT assays showed that in the cells expressing BTG2(APRO)boxC-HA, the poly(A) tails of the β-globin transcript were of similar length than in control cells and not shorter as observed in cells co-expressing the wild-type BTG2 APRO domain (Fig. 4d). The mutant protein was expressed at similar level as the wild-type protein, excluding that this observation resulted from protein destabilization by the mutation (Fig. 4d). Thus, the boxC BTG2 mutant that does not interact with PABPC1 RRMs but still interacts with CAF1 did not stimulate mRNA deadenylation *in cellulo*.

Altogether, the results demonstrated that the interaction of BTG2 with the first RRM of PABPC1 is an essential part of the mechanism by which BTG2 stimulates mRNA deadenylation.

### Reduced cell proliferation requires BTG2–PABPC1 interaction

**Figure 5:**
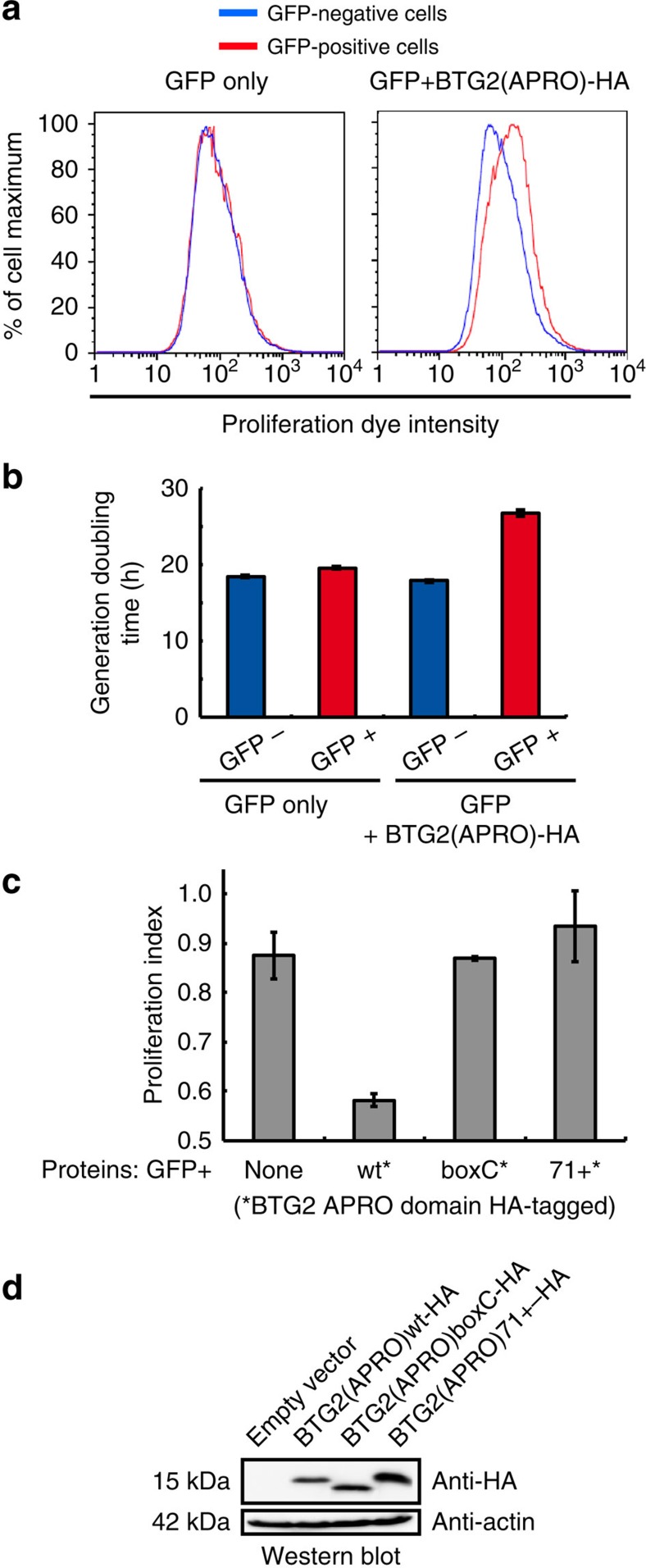
Interaction between BTG2 APRO and PABPC1 RRMs is necessary for BTG2 APRO domain to reduce cell proliferation. (**a**) Histograms showing the cell proliferation dye intensities in cells expressing or not BTG2(APRO). U2OS cells were labelled with the cell proliferation dye eFluor 670 before seeding in transfection plates. One day later, the cells were co-transfected with plasmids expressing GFP and BTG2(APRO)-HA (right panel) or GFP and empty expression vector (left panel), and were analysed 3 days after transfection using flow cytometry. (**b**) Histogram representing generation time of cells expressing or not the APRO domain of BTG2. U2OS cells were treated as in **a** and analysed 48, 72 and 96 h after transfection using flow cytometry. Half-life for the dilution of the mean proliferation dye intensity for the different cell populations was calculated from kinetic curves. These half-lives correlate directly to the cell-generation doubling time. Error bars (s.d.) correspond to two biological replicates. (**c**) Analysis of the ability of mutated BTG2 APRO domains to reduce cell proliferation. The analysis depicted in **a** was repeated with plasmids expressing WT, boxC-mutated (same mutation as in Fig. 3a) or 71+ insertion-mutant BTG2 APRO domains, or empty expression plasmid as control. Proliferation rate changes, quantified from the eFluor670 dye dilution, are plotted according to the formula: Proliferation index=1− [(mean dye intensity for GFP-positive cells–average of mean dye intensities for GFP-negative cells from all samples)/average of the mean dye intensities for GFP-negative cells from all samples]. Hence, a value of 1 corresponds to the proliferation of fast-growing GFP-negative cells, while an index of 0 indicates a reduction of one cell cycle division after transfection. Average (±s.d.) from two biological replicates. (**d**) A western blot analysis of cell lysates corresponding to the experiment presented in **c** is shown to monitor expression of the BTG2 proteins.

Having shown that the interaction between BTG2 and PABPC1 is necessary and sufficient to stimulate deadenylation *in vitro* and *in cellulo*, we next tested its implication in BTG2 antiproliferative activity. We first established a quantitative cellular proliferation assay using the Cell Proliferation Dye eFluor 670 (eBioscience). This fluorescent dye binds covalently to any cellular proteins containing primary amines and is distributed equally between daughters when cells divide. U2OS cells were pulse-labelled with eFluor and co-transfected 1 day later with two plasmids, one expressing GFP and the second BTG2(APRO)-HA (or as control, empty expression vector). Three days later, the cells were analysed using flow cytometry. The results showed that for cells co-transfected with GFP and empty expression plasmid, the peaks corresponding to the proliferation dye intensities of the GFP-negative (not transfected) and GFP-positive cells precisely overlap (Fig. 5a, left panel). This indicates that these two cell populations divided with the same kinetics. By contrast, for the cells co-transfected with plasmids expressing GFP and BTG2(APRO)-HA, the peak of dye intensities for the GFP-positive cells (co-expressing BTG2 and GFP) is shifted towards higher values as compared with the peak for the GFP-negative cells (Fig. 5a, right panel). This indicates that the cells expressing BTG2(APRO)-HA proliferated more slowly than the untransfected cells. Thus, expression of the APRO domain of BTG2 is sufficient to slow cell proliferation. Generation doubling time measurements indicated that, in these conditions, cells expressing the APRO domain of BTG2 had a doubling time increased by approximately 40% (Fig. 5b).

We then co-expressed the mutant boxC BTG2 APRO domain that still interacts with CAF1 and PRMT1 but not with the first RRM domain of PABPC1 (Fig. 4a and Supplementary Fig. 8a,b) with GFP in U2OS cells that had been labelled with the proliferation dye. We quantified cell proliferation for the different conditions from the average dye intensities of the GFP-negative and -positive cells (see Fig. 5c legend). This revealed that the mutated BTG2 APRO domain had little influence on cell proliferation in contrast to the expression of the wild-type protein (Fig. 5c,d). Thus, BTG2 binding to the RRM domains of PABPC1 is required for BTG2 to exert antiproliferative functions.

It has been published previously that the antiproliferative activity of BTG2 requires an interaction with CAF1 (ref. 44). The BTG2 W103A mutant used in this study was, however, not assessed for its interaction with PABPC1. As this mutant was not well expressed in our assays, we used instead a CAF1-interaction-defective BTG2 mutant that we selected previously from a transposon-based BTG2 mutant library^33^. This BTG2(APRO)71+ mutant results from an insertion of five amino acids after N71 and shows impaired interaction with CAF1 but not with PABPC1 (Supplementary Fig. 9). Expression of this mutant in U2OS cells did not affect cell proliferation (Fig. 5c,d). Thus, the antiproliferative property of BTG2 requires both interaction with CAF1 and PABPC1, which suggests strongly that this BTG2 property is due to its function in mRNA deadenylation.

## Discussion

We have shown previously that BTG2 is a general activator of mRNA deadenylation^33^. However, the mechanism by which expression of BTG2 stimulated mRNA deadenylation remained so far mysterious. The observations reported here are consistent with a mechanism by which BTG2 stimulates mRNA deadenylation by recruiting CAF1 deadenylase (and possibly associated partners) through its binding to the first RRM domain of PABPC1 bound to poly(A). This mechanism is probably valid for BTG1, as its APRO domain binds also to the first RRM domains of PABPC1.

Even though the mechanism by which BTG1 and BTG2 stimulate deadenylation shows similarities with the mechanism used by Tob proteins, the fact that different PABPC1 domains are involved has potentially important consequences. In particular, BTG1/BTG2 recruits CAF1 to the domains of PABPC1 that are also involved in poly(A) binding. Interestingly, structural studies have shown that poly(A) is orientated in such a manner that its 3′ end exits from PABPC1 first RRM domain, whereas its 5′ end is bound by the second RRM domain^45^. Furthermore, our mutagenesis study of BTG2 suggests that PABPC1 first RRM binds BTG2 at a surface close to the CAF1 catalytic cavity. It is thus possible that BTG2 bridges PABPC1 first RRM domain and CAF1 to allow more efficient delivery of the poly(A) 3′ extremity to the catalytic site of the deadenylase. Such mechanism might not occur for Tob proteins. Furthermore, as BTG2 APRO domain displays slightly higher affinity for PABPC1 compared with Tob proteins^37^, BTG2 might be a more effective activator of deadenylation than Tobs. Moreover, BTG1/2, but not Tob factors, would still be active in cells in which virally encoded proteases have truncated PABPC1, removing its MLLE domain^46^,^47^,^48^,^49^. In addition, the RRM domains of PABPC interact with proteins involved in translation initiation, such as the initiation factor eIF4G (ref. 50) and the translational regulators Paip1 and Paip2 (ref. 51). It would then be interesting to see whether BTG1/2 binding to the first RRM affects eIF4G, Paip1 or Paip2 binding (through sterical or allosteric effects) and thus translational regulation.

Importantly, we observed that the ability of the BTG2 APRO domain to interact with the first RRM domain of PABPC1, and to consequently stimulate deadenylation *in vivo* and *in vitro*, correlated with its antiproliferative properties. This further strengthens the idea that the antiproliferative properties of BTG/Tob proteins are linked to their function in mRNA deadenylation. In agreement with the fact that PABPC1 is a general mRNA-binding protein, ectopic expression of BTG2 and Tob was shown to stimulate the deadenylation of all the reporters or endogenous transcripts tested^5^,^33^. Tob has been reported since to interact with the cytoplasmic polyadenylation element-binding proteins (CPEB)^52^, suggesting that Tob could regulate the deadenylation of specific mRNA targets through a dual interaction with CPEB and PABPC. The c-myc mRNA has been identified as one of these specific targets and it was proposed that this could contribute to the mechanism through which Tob regulates cell growth^53^. In light of these data, it would be interesting to test whether endogenous expression of BTG2 affects also globally mRNA deadenylation, an activity that could be interesting to facilitate implementation of major changes in gene expression programmes. Alternatively, BTG2 may promote the degradation of specific groups of mRNAs that suppress proliferation and promote differentiation by acting synergistically with other mRNA-binding factors.

PABPC coating of the poly(A) tail is intuitively considered as protecting mRNA against degradation by exonucleases. Yet, the relation of PABPC with deadenylases is complex. PABPC has the ability to recruit the PAN2–PAN3 deadenylase complex^54^,^55^ through a direct interaction with PAN3 (ref. 56) and this in addition stimulates PAN2 deadenylase activity^57^. However, the exact impact of deadenylation by the PAN2–PAN3 complex on mRNA expression is poorly understood and the deletion of the corresponding genes in yeast has low influence on growth and poly(A) tail length^58^,^59^. The connection of PABPC with the other deadenylases is more ambiguous. Indeed, PABPC was shown to inhibit *in vitro* deadenylation in cell extracts^60^,^61^, and in certain conditions the activity of purified PARN or yeast CCR4 and CAF1 deadenylases^62^,^63^,^64^. PABPC was also shown to be part of protein complexes inhibiting deadenylation in cells^65^. Nevertheless, a mechanism allowing the recruitment of the CCR4–CAF1 deadenylases to mRNA through a direct interaction with PABPC evolved with the appearance during evolution of the BTG/Tob factors and we show here that PABPC1 plays an active role in stimulating CAF1 deadenylase activity together with BTG2. Our *in vitro* experiments indicate that this stimulation occurs in the absence of any additional factor. These observations bring one more step in the complexity of PABPC1 function in regulating mRNA poly(A) tail length and suggest that CAF1 deadenylase can be activated to deadenylate substrates when not integrated in the CCR4–NOT assembly.

## Methods

### Plasmid constructions

Plasmids used in this study are presented in Supplementary Data 1 and the sequences of the oligonucleotides in Supplementary Data 2. Segments amplified with PCR were verified by sequencing.

### Cell culture and transfections

Cell lines HEK293 (ATCC CRL-1573) and HEK293 Tet-Off (described in ref. 33) were maintained in DMEM medium containing 4.5 g l^−1^ glucose, GlutaMAX, 10% fetal calf serum and 40 μg ml^−1^ gentamycine. They were transfected with Effectene transfection reagent (Qiagen) as recommended by the manufacturer.

U2OS cells (ATCC HTB-96) were maintained in DMEM medium supplemented with 1 g l^−1^ glucose, 10% fetal calf serum and 40 μg ml^−1^ gentamycine. For the cell proliferation assays, U2OS cells were transfected with DharmaFECT Duo (Dharmacon) as recommended by the manufacturer.

### RNA extraction and RACE-PAT

Total RNA was extracted using the NucleoSpin RNA kit (Macherey-Nagel).

To monitor the length of the reporter β-globin transcript poly(A) tail, a modified RACE-PAT assay was used as described previously^66^. Briefly, a synthetic R2 RNA oligonucleotide with a phosphate group at its 5′ end and a dideoxyC nucleotide at its 3′ end was ligated to total cellular RNA with T4 RNA ligase 1 (New England Biolabs). After reverse transcription with oligonucleotide OBS4420, containing the complementary sequence of R2 oligonucleotide, PCR amplification was performed with a β-globin-specific primer, OBS4069, and oligonucleotide OBS4437 that is partially complementary to OBS4420. PCR products were resolved by migration on 3% agarose gels stained with ethidium bromide that were digitized using Typhoon 8600 (GE Healthcare).

### Protein co-immunoprecipitation and western blot

Cells were lysed in IPP150 lysis buffer (10 mM Tris-HCl pH 8.0, 150 mM NaCl, 1% Igepal CA-630 and protease inhibitors) by standard procedures.

For the co-precipitation experiments shown in Fig. 1d, cell lysates were incubated with monoclonal anti-HA agarose (Sigma) for 1 h at 4 °C. Beads were washed three times with IPP150 buffer (10 mM Tris-HCl pH 8.0, 150 mM NaCl, 0.1% Igepal CA-630) and eluted with Laemmli sample buffer (60 mM Tris-HCl pH 6.8, 10% glycerol, 0.002% bromophenol blue, 2% sodium dodecyl sulfate and 5% β-mercaptoethanol).

For the co-precipitation experiments shown in Fig. 2d, cells were treated 24 h after transfection with 0.25 mM Lomant's reagent (Dithio-bis Succinimidyl Propionate, Pierce) in PBS, complemented with 0.1 mM CaCl_2_ and 1 mM MgCl_2_, for 30 min at room temperature and quenched 15 min on ice in PBS–0.1 mM CaCl_2_–1 mM MgCl_2_ supplemented with 20 mM Tris-HCl pH 7.5. Cells were lysed for 30 min on ice in 200 μl of Triton-lysis buffer (10 mM Tris-HCl pH 7.5, 150 mM NaCl, 0.5 mM EDTA, 0.5% Triton X-100 and protease inhibitors) supplemented with 40 μg ml^−1^ RNAse A. After centrifugation, 300 μl of wash buffer (10 mM Tris-HCl pH 7.5, 150 mM NaCl and 0.5 mM EDTA) was added to the supernatant. After addition of 10 μl GFP-Trap matrix (ChromoTek), lysates were incubated for 1 h at 4 °C, beads were washed three times with wash buffer and were eluted 10 min at 95 °C with Laemmli sample buffer (60 mM Tris-HCl pH 6.8, 10% glycerol, 0.002% bromophenol blue, 2% sodium dodecyl sulfate and 5% β-mercaptoethanol).

Western blotting was performed by standard procedures and visualized with ImageQuant LAS4000 (GE Healthcare). The HA tag, GFP, PABPC1 and CNOT7 were revealed with monoclonal antibody anti-HA (HA.11, COVANCE # MMS-101P), monoclonal antibody anti-GFP (JL-8, Clontech # 632381), monoclonal antibody anti-PABPC1 (10E10, Sigma # P 6246) and a rabbit antiserum anti-CNOT7 generated in the laboratory. Monoclonal antibodies anti-β-tubulin (2A2) and anti-α-actin (2D7) were generated at the IGBMC. All the antibodies were used at a 1–1,000 dilution. The SuperSignal West Femto Maximum Sensivity Substrate kit (Pierce) or Luminata Crescendo Western HRP Substrate were used as HRP substrates. For the low-affinity anti-CNOT7 antiserum, the SuperSignal Western Blot Enhancer kit (Pierce) was used to enhance the signal. The full western blots are shown in Supplementary Fig. 10.

### Yeast two-hybrid screen and assays

A two-hybrid screen of a human fibroblast cDNA library with a LexA-Binding-Domain-BTG2(APRO) fusion bait was performed by Hybrigenics. To test pairwise interactions, the diploid yeast strain Y187/L40 (*MATα/MATa, ade2-101/ade2, his3-200/his3*Δ*200, leu2-3,-112/leu2-3,-112, lys2-801am::LYS2::(lexAop)4-HIS3/LYS2, MEL1/?, met-/MET, trp1-901/trp1-901, gal4Δ/gal4-542, gal80*Δ*/gal80-538, ura3-52::URA3::GAL1UAS-GAL1TATA-lacZ/URA3::(lexAop)8-lacZ*) was transformed simultaneously with the LexA-Binding-Domain and Gal4-Activating-Domain-derived plasmids by standard LiAc procedures. Interaction between the different chimeric proteins was monitored by β-galactosidase assays performed using the Beta-Glo Assay System (Promega) as recommended by the manufacturer.

### His- or GST-PD experiments

Proteins were co-expressed in *E. coli* BL21-CodonPlus strain (Stratagene) grown in autoinduction media (Formedium). Bacteria were lysed by sonication in binding buffers (for His-PD: 50 mM Tris-HCl pH 8.0, 40 mM Imidazole, 300 mM NaCl, 1 mM β-mercaptoethanol and 0.1% Igepal; for GST-PD: PBS) supplemented with protease inhibitors (Complete Protease Inhibitor Cocktail EDTA-free, Roche). After centrifugation to remove insoluble material, lysates were incubated for 1 h at 4 °C with the following: for His-PD, Ni-NTA resin (Qiagen) and for GST-PD, Glutathione Sepharose 4B resin (GE Healthcare Life Sciences). After centrifugation at 500*g* for 1 min, the beads were washed twice with 1 ml binding buffer and eluted in the following: for His-PD, 50 mM Tris-HCl pH 8.0, 200 mM Imidazole, 300 mM NaCl; for GST-PD, 10 mM reduced glutathione, 50 mM Tris-HCl pH 8.0.

### Purification of recombinant proteins

Recombinant proteins were produced in *E. coli* BL21-CodonPlus strain (Stratagene) grown in autoinduction media (Formedium).

*GST, GST-BTG2(APRO) and GST-Tob1(APRO)* (Fig. 3d): Bacteria were lysed in PBS1X buffer supplemented with protease inhibitors (Complete Protease Inhibitor Cocktail EDTA-free, Roche) using a Cell Disrupter Device (Constant Systems Ltd). GST-tagged proteins were bound to Glutathione Sepharose 4B resin (GE Healthcare Life Sciences) in Poly-Prep column (Bio-Rad), eluted with 10 mM reduced glutathione in 50 mM Tris-HCl pH 8.0, dialysed and stored in PBS 1X.

*GST, GST-BTG2(APRO)wt and GST-BTG2(APRO)boxC* (Fig. 4b). Bacteria were lysed by sonication in buffer (30 mM HEPES-KOH pH 7.5, 100 mM NaCl, 1 mM CHAPS and 2 mM dithiothreitol (DTT)) supplemented with protease inhibitors (Complete Protease Inhibitor Cocktail EDTA-free, Roche) and Benzonase (BaseMuncher, Expedeon). GST-tagged proteins were purified with affinity chromatography on GSTrap FF column and then on Sephacryl S200 16/50 column (GE Healthcare Life Sciences) with PBS 1X and stored in PBS 1X.

*BTG2(APRO)*. Bacteria producing GST-BTG2(APRO) were resuspended in lysis buffer (30 mM HEPES-KOH pH 7.5, 100 mM NaCl, 1 mM CHAPS and 1 mM DTT) supplemented with protease inhibitors (Complete Protease Inhibitor Cocktail EDTA-free, Roche) and Benzonase (BaseMuncher, Expedeon) and lysed by sonication. GST-BTG2(APRO) was purified with affinity chromatography on GSTrap FF column (GE Healthcare Life Sciences), the GST tag was removed by thrombin cleavage and BTG2(APRO) was further purified with a size exclusion chromatography step on Superdex 75 column (GE Healthcare Life Sciences) in 20 mM HEPES-KOH pH 7.5, 50 mM NaCl and 0.5 mM TCEP.

*6His-PABPC1(1–190) and 6His-CNOT7*. Bacteria were resuspended in lysis buffer (30 mM HEPES-KOH pH 7.5, 300 mM NaCl and 1 mM DTT) supplemented with protease inhibitors (Complete Protease Inhibitor Cocktail EDTA-free, Roche) and Benzonase (BaseMuncher, Expedeon) and lysed by sonication. Proteins were purified with affinity chromatography on HisTrap FF crude column (GE Healthcare Life Sciences) followed by a size exclusion chromatography step on Superdex 75 column (GE Healthcare Life Sciences). Proteins were stored in gel filtration buffer containing 20 mM HEPES-KOH pH 7.5, 50 mM NaCl and 0.5 mM TCEP.

*6His-PABPC1(FL)*. Bacteria were lysed in PBS1X buffer supplemented with 20 mM Imidazole, 350 mM NaCl and protease inhibitors (Complete Protease Inhibitor Cocktail EDTA-free, Roche) using a Cell Disrupter Device (Constant Systems Ltd.). 6His-PABPC1(FL) was purified using affinity chromatography on HisTrap FF column (GE Healthcare Life Sciences) followed by a size exclusion chromatography step on Superdex 75 column (GE Healthcare Life Sciences) in PBS1X buffer. 6His-PABPC1(FL) was stored in PBS1X buffer.

### *In vitro* deadenylation assays

A poly(A) substrate possessing approximately 75 residues was obtained by extending a synthetic 20(A) RNA oligonucleotide labelled with Fluorescein at its 5′ end (Dharmacon) with ATP and *E. coli* Poly(A) Polymerase (New England BioLabs). The substrate of desired length was purified by denaturing polyacrylamide gel electrophoresis.

Electrophoretic mobility shift assays (Supplementary Methods) determined that 7 picomoles of the purified 6His-PABPC1(FL) or 6His-PABPC1(1–190) proteins were necessary to shift completely 1 picomoles of the fluorescently labelled poly(A) substrate (Supplementary Fig. 4a,b). We performed then *in vitro* deadenylation assays in 10 μl final volume using 1 picomoles of the poly(A) substrate with 7 picomoles of 6His-PABPC1(FL) or 6His-PABPC1(1–190) proteins, 18 picomoles of BTG2(APRO) or GST, GST-BTG2(APRO), GST-Tob1(APRO) fusion proteins (an excess of BTG2 proteins to PABPC1 was used to favour formation of the BTG2/PABPC1 complex) and 18 picomoles of 6His-CNOT7 (in equimolar amount as compared with the BTG2 proteins), unless otherwise indicated. Reactions were performed at 30 °C in buffer containing 20 mM HEPES-KOH pH 8.0; 100 mM KCl; 0.5 mM DTT; 0.1% Igepal CA-630; 5 mM MgAc. At indicated time, one aliquot of the reaction was mixed with an equal volume of RNA dye buffer (100 mM Tris-HCl pH 7.5; 1 mM EDTA; 0.001% bromophenol blue; 0.001% xylene cyanol; 0.05% sodium dodecyl sulfate; and 92% formamide) and kept on ice. Products were analysed by electrophoresis on 8 or 15% denaturing polyacrylamide gel and visualized with ImageQuant LAS4000 or Typhoon 8600 (GE Healthcare). Nuclease P1 (Sigma) was used as recommended by the manufacturer.

### Gel quantification

The fluorescence intensity profile of each lane was acquired with the ImageQuant software (GE Healthcare). Each profile was converted into a numerical table using the Engauge Digitizer software. The intensity-weighted average RNA size (calculated in nucleotide by comparison with molecular size markers) was determined for regions with signal intensities above a minimal threshold after background subtraction. For evaluation of the average poly(A) tail length indicated in Fig. 1b legend, the size of the poly(A) minus control was subtracted. Fitting a straight line for average RNA length as a function of time determined the deadenylation rate for the *in vitro* deadenylation assays.

### Cell proliferation assay

U2OS cells, labelled with the Cell Proliferation Dye eFluor 670 (eBioscience) as recommended by the manufacturer, were seeded in 12-well plates to reach ∼90% confluence 24 h later at which time they were transiently co-transfected with a plasmid expressing GFP and plasmids expressing the BTG/Tob proteins using DharmaFECT Duo (Dharmacon) as transfection reagent. Twenty-four hours after transfection, they were trypsinized and seeded in larger plates. Seventy-two hours after transfection (or 48, 72 and 96 h after transfection for proliferation rate measurement), they were trypsinized and resuspended in PBS buffer. After addition of propidium iodide to a final concentration of 0.5 μg ml^−1^, cells were analysed with flow cytometry using a BD FACSCalibur flow cytometer.

## Additional information

**How to cite this article:** Stupfler, B. *et al*. BTG2 bridges PABPC1 RNA-binding domains and CAF1 deadenylase to control cell proliferation. *Nat. Commun.* 7:10811 doi: 10.1038/ncomms10811 (2016).

## Supplementary Material

Supplementary Information Supplementary Figures 1-10, Supplementary Methods and Supplementary Reference

Supplementary Data 1 List of plasmids used in this study

Supplementary Data 2 List of oligonuleotides used in this study

## References

[b1] Matsuda S., Rouault J., Magaud J. & Berthet C. In search of a function for the TIS21/PC3/BTG1/TOB family. FEBS Lett. 497, 67–72 (2001). 11377414 10.1016/s0014-5793(01)02436-x

[b2] Lim N. S. . Comparative peptide binding studies of the PABC domains from the ubiquitin-protein isopeptide ligase HYD and poly(A)-binding protein. Implications for HYD function. J. Biol. Chem. 281, 14376–14382 (2006). 16554297 10.1074/jbc.M600307200

[b3] Okochi K., Suzuki T., Inoue J., Matsuda S. & Yamamoto T. Interaction of anti-proliferative protein Tob with poly(A)-binding protein and inducible poly(A)-binding protein: implication of Tob in translational control. Genes Cells 10, 151–163 (2005). 15676026 10.1111/j.1365-2443.2005.00826.x

[b4] Funakoshi Y. . Mechanism of mRNA deadenylation: evidence for a molecular interplay between translation termination factor eRF3 and mRNA deadenylases. Genes Dev. 21, 3135–3148 (2007). 18056425 10.1101/gad.1597707 PMC2081979

[b5] Ezzeddine N. . Human TOB, an antiproliferative transcription factor, is a poly(A)-binding protein-dependent positive regulator of cytoplasmic mRNA deadenylation. Mol. Cell Biol. 27, 7791–7801 (2007). 17785442 10.1128/MCB.01254-07 PMC2169145

[b6] Winkler G. S. The mammalian anti-proliferative BTG/Tob protein family. J. Cell Physiol. 222, 66–72 (2010). 19746446 10.1002/jcp.21919

[b7] Iacopetti P., Barsacchi G., Tirone F., Maffei L. & Cremisi F. Developmental expression of PC3 gene is correlated with neuronal cell birthday. Mech. Dev. 47, 127–137 (1994). 7811636 10.1016/0925-4773(94)90085-x

[b8] Iacopetti P. . Expression of the antiproliferative gene TIS21 at the onset of neurogenesis identifies single neuroepithelial cells that switch from proliferative to neuron-generating division. Proc. Natl Acad. Sci. USA 96, 4639–4644 (1999). 10200315 10.1073/pnas.96.8.4639 PMC16385

[b9] Attardo A. . Tis21 expression marks not only populations of neurogenic precursor cells but also new postmitotic neurons in adult hippocampal neurogenesis. Cereb. Cortex 20, 304–314 (2010). 19482889 10.1093/cercor/bhp100 PMC2803732

[b10] Canzoniere D. . Dual control of neurogenesis by PC3 through cell cycle inhibition and induction of Math1. J. Neurosci. 24, 3355–3369 (2004). 15056715 10.1523/JNEUROSCI.3860-03.2004 PMC6730030

[b11] Fei J. F., Haffner C. & Huttner W. B. 3′ UTR-dependent, miR-92-mediated restriction of Tis21 expression maintains asymmetric neural stem cell division to ensure proper neocortex size. Cell Rep. 7, 398–411 (2014). 24726360 10.1016/j.celrep.2014.03.033

[b12] Farioli-Vecchioli S. . Impaired terminal differentiation of hippocampal granule neurons and defective contextual memory in PC3/Tis21 knockout mice. PLoS ONE 4, e8339 (2009). 20020054 10.1371/journal.pone.0008339 PMC2791842

[b13] Park S. . B-cell translocation gene 2 (Btg2) regulates vertebral patterning by modulating bone morphogenetic protein/smad signaling. Mol. Cell Biol. 24, 10256–10262 (2004). 15542835 10.1128/MCB.24.23.10256-10262.2004 PMC529031

[b14] Duriez C. . The human BTG2/TIS21/PC3 gene: genomic structure, transcriptional regulation and evaluation as a candidate tumor suppressor gene. Gene 282, 207–214 (2002). 11814693 10.1016/s0378-1119(01)00825-3

[b15] Boiko A. D. . A systematic search for downstream mediators of tumor suppressor function of p53 reveals a major role of BTG2 in suppression of Ras-induced transformation. Genes Dev. 20, 236–252 (2006). 16418486 10.1101/gad.1372606 PMC1356114

[b16] Kawakubo H. . Expression of the NF-kappaB-responsive gene BTG2 is aberrantly regulated in breast cancer. Oncogene 23, 8310–8319 (2004). 15378000 10.1038/sj.onc.1208008

[b17] Struckmann K. . Impaired expression of the cell cycle regulator BTG2 is common in clear cell renal cell carcinoma. Cancer Res. 64, 1632–1638 (2004). 14996721 10.1158/0008-5472.can-03-1687

[b18] Kawakubo H. . Loss of B-cell translocation gene-2 in estrogen receptor-positive breast carcinoma is associated with tumor grade and overexpression of cyclin d1 protein. Cancer Res. 66, 7075–7082 (2006). 16849553 10.1158/0008-5472.CAN-06-0379

[b19] Mollerstrom E. . Up-regulation of cell cycle arrest protein BTG2 correlates with increased overall survival in breast cancer, as detected by immunohistochemistry using tissue microarray. BMC Cancer 10, 296 (2010). 20553615 10.1186/1471-2407-10-296 PMC2902444

[b20] Xu K., Bai Y., Zhang A., Zhang Q. & Bartlam M. G. Insights into the structure and architecture of the CCR4-NOT complex. Front Genet. 5, 137 (2014). 24904637 10.3389/fgene.2014.00137 PMC4032980

[b21] Basquin J. . Architecture of the nuclease module of the yeast Ccr4-not complex: the Not1-Caf1-Ccr4 interaction. Mol. Cell 48, 207–218 (2012). 22959269 10.1016/j.molcel.2012.08.014

[b22] Tucker M. . The transcription factor associated Ccr4 and Caf1 proteins are components of the major cytoplasmic mRNA deadenylase in *Saccharomyces cerevisiae*. Cell 104, 377–386 (2001). 11239395 10.1016/s0092-8674(01)00225-2

[b23] Daugeron M. C., Mauxion F. & Seraphin B. The yeast POP2 gene encodes a nuclease involved in mRNA deadenylation. Nucleic Acids Res. 29, 2448–2455 (2001). 11410650 10.1093/nar/29.12.2448 PMC55743

[b24] Yamashita A. . Concerted action of poly(A) nucleases and decapping enzyme in mammalian mRNA turnover. Nat. Struct. Mol. Biol. 12, 1054–1063 (2005). 16284618 10.1038/nsmb1016

[b25] Lau N. C. . Human Ccr4-Not complexes contain variable deadenylase subunits. Biochem. J. 422, 443–453 (2009). 19558367 10.1042/BJ20090500

[b26] Fabian M. R. . Structural basis for the recruitment of the human CCR4-NOT deadenylase complex by tristetraprolin. Nat. Struct. Mol. Biol. 20, 735–739 (2013). 23644599 10.1038/nsmb.2572 PMC4811204

[b27] Bhandari D., Raisch T., Weichenrieder O., Jonas S. & Izaurralde E. Structural basis for the Nanos-mediated recruitment of the CCR4-NOT complex and translational repression. Genes Dev. 28, 888–901 (2014). 24736845 10.1101/gad.237289.113 PMC4003280

[b28] Mathys H. . Structural and biochemical insights to the role of the CCR4-NOT complex and DDX6 ATPase in microRNA repression. Mol. Cell 54, 751–765 (2014). 24768538 10.1016/j.molcel.2014.03.036

[b29] Chen Y. . A DDX6-CNOT1 complex and W-binding pockets in CNOT9 reveal direct links between miRNA target recognition and silencing. Mol. Cell 54, 737–750 (2014). 24768540 10.1016/j.molcel.2014.03.034

[b30] Guardavaccaro D. . Arrest of G(1)-S progression by the p53-inducible gene PC3 is Rb dependent and relies on the inhibition of cyclin D1 transcription. Mol. Cell Biol. 20, 1797–1815 (2000). 10669755 10.1128/mcb.20.5.1797-1815.2000 PMC85361

[b31] Prevot D. . The leukemia-associated protein Btg1 and the p53-regulated protein Btg2 interact with the homeoprotein Hoxb9 and enhance its transcriptional activation. J. Biol. Chem. 275, 147–153 (2000). 10617598 10.1074/jbc.275.1.147

[b32] Passeri D. . Btg2 enhances retinoic acid-induced differentiation by modulating histone H4 methylation and acetylation. Mol. Cell Biol. 26, 5023–5032 (2006). 16782888 10.1128/MCB.01360-05 PMC1489145

[b33] Mauxion F., Faux C. & Seraphin B. The BTG2 protein is a general activator of mRNA deadenylation. EMBO J. 27, 1039–1048 (2008). 18337750 10.1038/emboj.2008.43 PMC2323266

[b34] Ezzeddine N., Chen C. Y. & Shyu A. B. Evidence providing new insights into TOB-promoted deadenylation and supporting a link between TOB's deadenylation-enhancing and antiproliferative activities. Mol. Cell Biol. 32, 1089–1098 (2012). 22252318 10.1128/MCB.06370-11 PMC3295015

[b35] Sasajima H., Nakagawa K. & Yokosawa H. Antiproliferative proteins of the BTG/Tob family are degraded by the ubiquitin-proteasome system. Eur. J. Biochem. 269, 3596–3604 (2002). 12135500 10.1046/j.1432-1033.2002.03052.x

[b36] Hong J. W., Ryu M. S. & Lim I. K. Phosphorylation of serine 147 of tis21/BTG2/pc3 by p-Erk1/2 induces Pin-1 binding in cytoplasm and cell death. J. Biol. Chem. 280, 21256–21263 (2005). 15788397 10.1074/jbc.M500318200

[b37] Xie J., Kozlov G. & Gehring K. The ‘tale' of poly(A) binding protein: The MLLE domain and PAM2-containing proteins. Biochim. Biophys. Acta 1839, 1062–1068 (2014). 25120199 10.1016/j.bbagrm.2014.08.001

[b38] Kuhn U. & Pieler T. Xenopus poly(A) binding protein: functional domains in RNA binding and protein-protein interaction. J. Mol. Biol. 256, 20–30 (1996). 8609610 10.1006/jmbi.1996.0065

[b39] Melo E. O., Dhalia R., Martins de Sa C., Standart N. & de Melo Neto O. P. Identification of a C-terminal poly(A)-binding protein (PABP)-PABP interaction domain: role in cooperative binding to poly (A) and efficient cap distal translational repression. J. Biol. Chem. 278, 46357–46368 (2003). 12952955 10.1074/jbc.M307624200

[b40] Lin J., Fabian M., Sonenberg N. & Meller A. Nanopore detachment kinetics of poly(A) binding proteins from RNA molecules reveals the critical role of C-terminus interactions. Biophys. J. 102, 1427–1434 (2012). 22455926 10.1016/j.bpj.2012.02.025 PMC3309291

[b41] Yang X. . Crystal structures of human BTG2 and mouse TIS21 involved in suppression of CAF1 deadenylase activity. Nucleic Acids Res. 36, 6872–6881 (2008). 18974182 10.1093/nar/gkn825 PMC2588512

[b42] Berthet C. . Interaction of PRMT1 with BTG/TOB proteins in cell signalling: molecular analysis and functional aspects. Genes Cells 7, 29–39 (2002). 11856371 10.1046/j.1356-9597.2001.00497.x

[b43] Horiuchi M. . Structural basis for the antiproliferative activity of the Tob-hCaf1 complex. J. Biol. Chem. 284, 13244–13255 (2009). 19276069 10.1074/jbc.M809250200 PMC2676056

[b44] Doidge R., Mittal S., Aslam A. & Winkler G. S. The anti-proliferative activity of BTG/TOB proteins is mediated via the Caf1a (CNOT7) and Caf1b (CNOT8) deadenylase subunits of the Ccr4-Not complex. PLoS ONE 7, e51331 (2012). 23236473 10.1371/journal.pone.0051331 PMC3517456

[b45] Deo R. C., Bonanno J. B., Sonenberg N. & Burley S. K. Recognition of polyadenylate RNA by the poly(A)-binding protein. Cell 98, 835–845 (1999). 10499800 10.1016/s0092-8674(00)81517-2

[b46] Kerekatte V. . Cleavage of Poly(A)-binding protein by coxsackievirus 2A protease *in vitro* and *in vivo*: another mechanism for host protein synthesis shutoff? J. Virol. 73, 709–717 (1999). 9847377 10.1128/jvi.73.1.709-717.1999 PMC103878

[b47] Alvarez E., Castello A., Menendez-Arias L. & Carrasco L. HIV protease cleaves poly(A)-binding protein. Biochem. J. 396, 219–226 (2006). 16594896 10.1042/BJ20060108 PMC1462710

[b48] Zhang B., Morace G., Gauss-Muller V. & Kusov Y. Poly(A) binding protein, C-terminally truncated by the hepatitis A virus proteinase 3C, inhibits viral translation. Nucleic Acids Res. 35, 5975–5984 (2007). 17726047 10.1093/nar/gkm645 PMC2034478

[b49] Kobayashi M., Arias C., Garabedian A., Palmenberg A. C. & Mohr I. Site-specific cleavage of the host poly(A) binding protein by the encephalomyocarditis virus 3C proteinase stimulates viral replication. J. Virol. 86, 10686–10694 (2012). 22837200 10.1128/JVI.00896-12 PMC3457283

[b50] Safaee N. . Interdomain allostery promotes assembly of the poly(A) mRNA complex with PABP and eIF4G. Mol. Cell. 48, 375–386 (2012). 23041282 10.1016/j.molcel.2012.09.001

[b51] Derry M. C., Yanagiya A., Martineau Y. & Sonenberg N. Regulation of poly(A)-binding protein through PABP-interacting proteins. Cold Spring Harb. Symp. Quant. Biol. 71, 537–543 (2006). 17381337 10.1101/sqb.2006.71.061

[b52] Hosoda N. . Anti-proliferative protein Tob negatively regulates CPEB3 target by recruiting Caf1 deadenylase. EMBO J. 30, 1311–1323 (2011). 21336257 10.1038/emboj.2011.37 PMC3094127

[b53] Ogami K., Hosoda N., Funakoshi Y. & Hoshino S. Antiproliferative protein Tob directly regulates c-myc proto-oncogene expression through cytoplasmic polyadenylation element-binding protein CPEB. Oncogene 33, 55–64 (2014). 23178487 10.1038/onc.2012.548

[b54] Christie M., Boland A., Huntzinger E., Weichenrieder O. & Izaurralde E. Structure of the PAN3 pseudokinase reveals the basis for interactions with the PAN2 deadenylase and the GW182 proteins. Mol. Cell 51, 360–373 (2013). 23932717 10.1016/j.molcel.2013.07.011

[b55] Schafer I. B., Rode M., Bonneau F., Schussler S. & Conti E. The structure of the Pan2-Pan3 core complex reveals cross-talk between deadenylase and pseudokinase. Nat. Struct. Mol. Biol. 21, 591–598 (2014). 24880344 10.1038/nsmb.2834

[b56] Siddiqui N. . Poly(A) nuclease interacts with the C-terminal domain of polyadenylate-binding protein domain from poly(A)-binding protein. J. Biol. Chem. 282, 25067–25075 (2007). 17595167 10.1074/jbc.M701256200

[b57] Wolf J. . Structural basis for Pan3 binding to Pan2 and its function in mRNA recruitment and deadenylation. EMBO J. 33, 1514–1526 (2014). 24872509 10.15252/embj.201488373 PMC4158885

[b58] Boeck R. . The yeast Pan2 protein is required for poly(A)-binding protein-stimulated poly(A)-nuclease activity. J. Biol. Chem. 271, 432–438 (1996). 8550599 10.1074/jbc.271.1.432

[b59] Brown C. E., Tarun S. Z. Jr, Boeck R. & Sachs A. B. PAN3 encodes a subunit of the Pab1p-dependent poly(A) nuclease in *Saccharomyces cerevisiae*. Mol. Cell Biol. 16, 5744–5753 (1996). 8816488 10.1128/mcb.16.10.5744 PMC231575

[b60] Bernstein P., Peltz S. W. & Ross J. The poly(A)-poly(A)-binding protein complex is a major determinant of mRNA stability *in vitro*. Mol. Cell Biol. 9, 659–670 (1989). 2565532 10.1128/mcb.9.2.659-670.1989 PMC362643

[b61] Wang Z., Day N., Trifillis P. & Kiledjian M. An mRNA stability complex functions with poly(A)-binding protein to stabilize mRNA *in vitro*. Mol. Cell. Biol. 19, 4552–4560 (1999). 10373504 10.1128/mcb.19.7.4552 PMC84253

[b62] Korner C. G. . The deadenylating nuclease (DAN) is involved in poly(A) tail removal during the meiotic maturation of Xenopus oocytes. EMBO J. 17, 5427–5437 (1998). 9736620 10.1093/emboj/17.18.5427 PMC1170868

[b63] Tucker M., Staples R. R., Valencia-Sanchez M. A., Muhlrad D. & Parker R. Ccr4p is the catalytic subunit of a Ccr4p/Pop2p/Notp mRNA deadenylase complex in *Saccharomyces cerevisiae*. EMBO J. 21, 1427–1436 (2002). 11889048 10.1093/emboj/21.6.1427 PMC125913

[b64] Simon E. & Seraphin B. A specific role for the C-terminal region of the Poly(A)-binding protein in mRNA decay. Nucleic Acids Res. 35, 6017–6028 (2007). 17766253 10.1093/nar/gkm452 PMC2094065

[b65] Grosset C. . A mechanism for translationally coupled mRNA turnover: interaction between the poly(A) tail and a c-fos RNA coding determinant via a protein complex. Cell 103, 29–40 (2000). 11051545 10.1016/s0092-8674(00)00102-1

[b66] Mauxion F., Preve B. & Seraphin B. C2ORF29/CNOT11 and CNOT10 form a new module of the CCR4-NOT complex. RNA Biol. 10, 267–276 (2013). 23232451 10.4161/rna.23065 PMC3594285

