# Supplementary material for: BTG2 bridges PABPC1 RNA-binding domains and CAF1 deadenylase to control cell proliferation
Source: Nat Commun. 2016 Feb 25;7:10811. doi: 10.1038/ncomms10811 (PMC4773420; doi:10.1038/ncomms10811)
Supplement: Supplementary Information — Supplementary Figures 1-10, Supplementary Methods and Supplementary Reference [file ncomms10811-s1.pdf]

SUPPLEMENTARY FIGURES

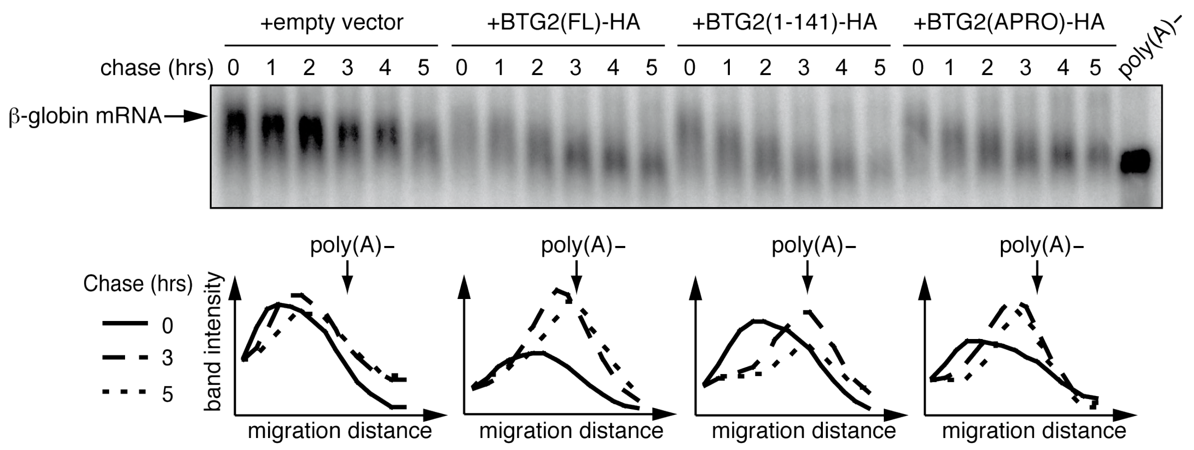

Supplementary Figure 1: BTG2 APRO domain is sufficient to stimulate deadenylation of the β-globin reporter transcript.

HEK293 Tet-Off cells were co-transfected with a plasmid expressing the β-globin reporter and plasmids expressing full-length (FL) or HA-tagged BTG2 proteins progressively truncated until only the APRO domain was left (amino acids 1 to 126), or with empty expression vector. Deadenylation kinetic of the reporter was visualized by transcriptional pulse-chase experiment followed by RNA extraction and Northern blotting. An RNA sample treated with oligo(dT) and RNase H was used as a marker for the migration of the fully deadenylated β-globin mRNA (poly(A)–). The profiles of mRNA migration for the 0, 3 and 5 hours chase times are shown (other times not shown in order not to saturate the graphs). The Northern blot image was quantified as described in the Supplementary Methods.

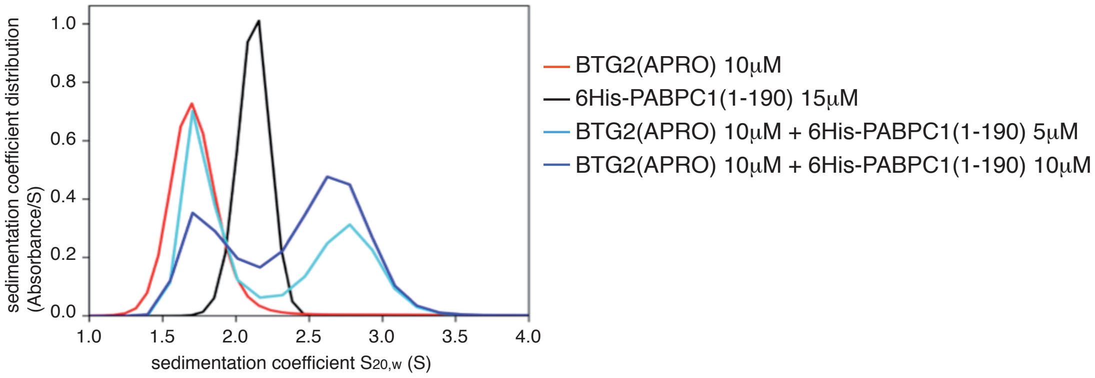

Supplementary Figure 2: Sedimentation velocity analysis showing the formation of a complex between the APRO domain of BTG2 and the first RRM domains of PABPC1 in the absence of RNA.

Bacterial pellets expressing BTG2(APRO) and 6His-PABPC1(1-190) proteins were lysed in the presence of Benzonase and the proteins were purified to homogeneity by affinity and size exclusion chromatography. Absorbance monitoring during the purifications did not reveal the presence of significant quantities of nucleic acids associated with the proteins. The purified proteins were then mixed in indicated ratio, in the absence of RNA. Analytical ultracentrifugation analyses indicate that BTG2(APRO) and 6His-PABPC1(1-190) are monomeric (peaks at 1.72 S and 2.12 S with estimated molecular masses of 15.3 kDa and 20.2 kDa respectively for calculated masses of 14.2 kDa and 22.4 kDa) and form 1:1 complexes when mixed together (new peak at 2.65 S with estimated molecular mass of 26.2 kDa. The mass is underestimated due to incomplete complex formation and/or cycles of association/dissociation).

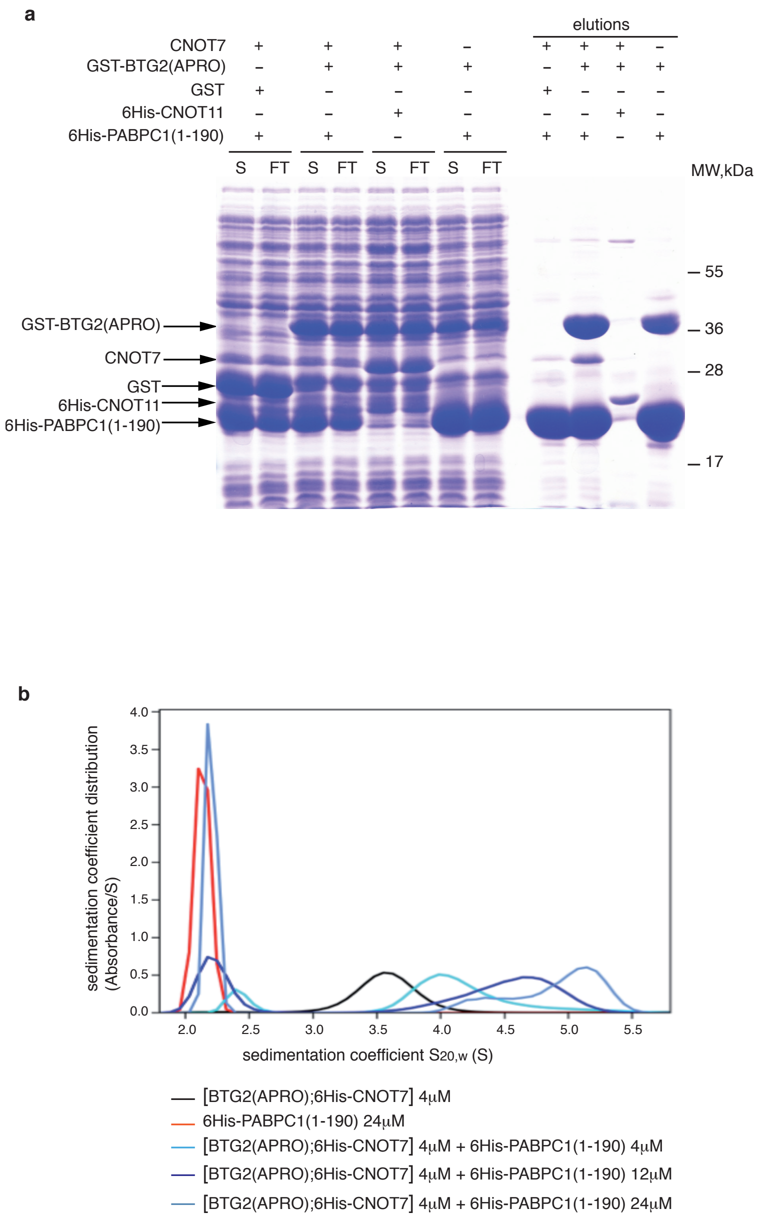

Supplementary Figure 3: Simultaneous binding of CAF1 (CNOT7 paralog) and PABPC1 to BTG2(APRO).

(**a**) – Co-purification of BTG2(APRO) and CNOT7 with PABPC1. 6His-PABPC1(1-190), or 6His-CNOT11 as negative control, were co-expressed in bacteria with GST or GST-BTG2(APRO) in the presence or not of untagged CNOT7, as indicated. 6His-tagged proteins

were purified on Ni agarose resin and fractionated with co-purifying factors on 12% SDS-PAGE gel stained with Coomassie blue.

(b) – Sedimentation velocity analysis showing the formation of a complex between the APRO domain of BTG2, the first RRM domains of PABPC1 and CNOT7. GST-BTG2(APRO) and 6His-CNOT7 were co-expressed in bacteria. The [BTG2(APRO);6His-CNOT7] complex was purified by affinity chromatography on His-trap column, followed by removal of the GST tag by thrombin cleavage and capture on a GSTrap column. 6His-PABPC1(1-190) was purified by affinity chromatography on His-trap column followed by size exclusion chromatography. [BTG2(APRO);6His-CNOT7] was mixed with 6His-PABPC1(1-190) in the indicated ratio. Sedimentation coefficients of the proteins were determined by analytical ultracentrifugation: 6His-PABPC1(1-190) alone peaks at 2.13 S with estimated molecular mass of 19 kDa (calculated molecular mass: 22.4 kDa); [BTG2(APRO);6His-CNOT7] peaks at 3.58 S with estimated molecular mass of 48.1 kDa (calculated molecular mass: 49.3 kDa) indicative of 1:1 complexes. When the three proteins are mixed together, peaks of higher sedimentation coefficients appear indicative of the formation of complexes between the three proteins. Peaks of increasing sedimentation coefficients were observed with increasing quantities of 6His-PABPC1(1-190), indicative that formation of a homogenous stoichiometric complex was not achieved. In these conditions, the mass of the heterotrimeric complex could thus not be determined.

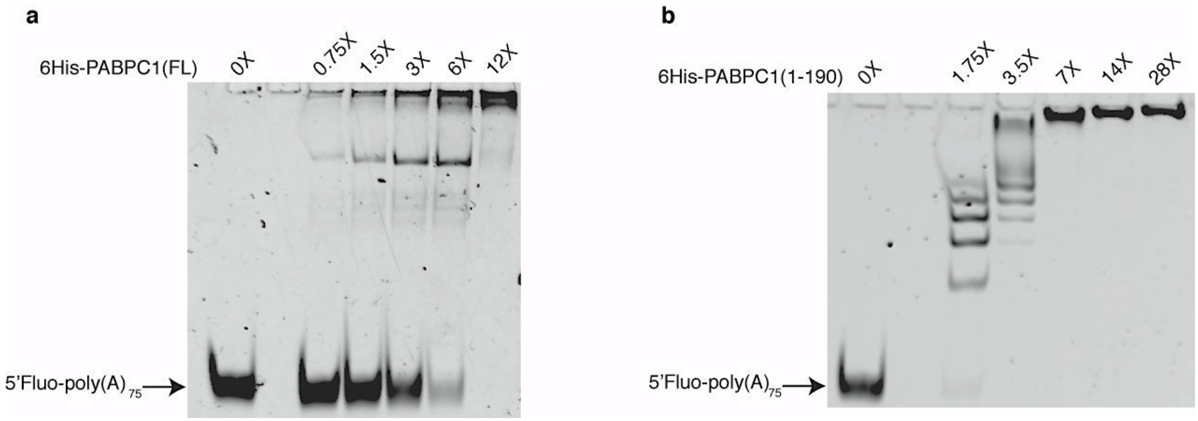

Supplementary Figure 4: PABPC1 proteins binding to the 5'Fluo-poly(A)$_{75}$ substrate.

The 5'-fluorescein-labeled RNA substrate (100nM) was incubated with increasing amount of purified 6His-PABPC1(FL) **(a)** or 6His-PABPC1(1-190) **(b)** as indicated (fold excess compare to substrate) and electrophoresed on native polyacrylamide gels, as described in Supplementary Methods.

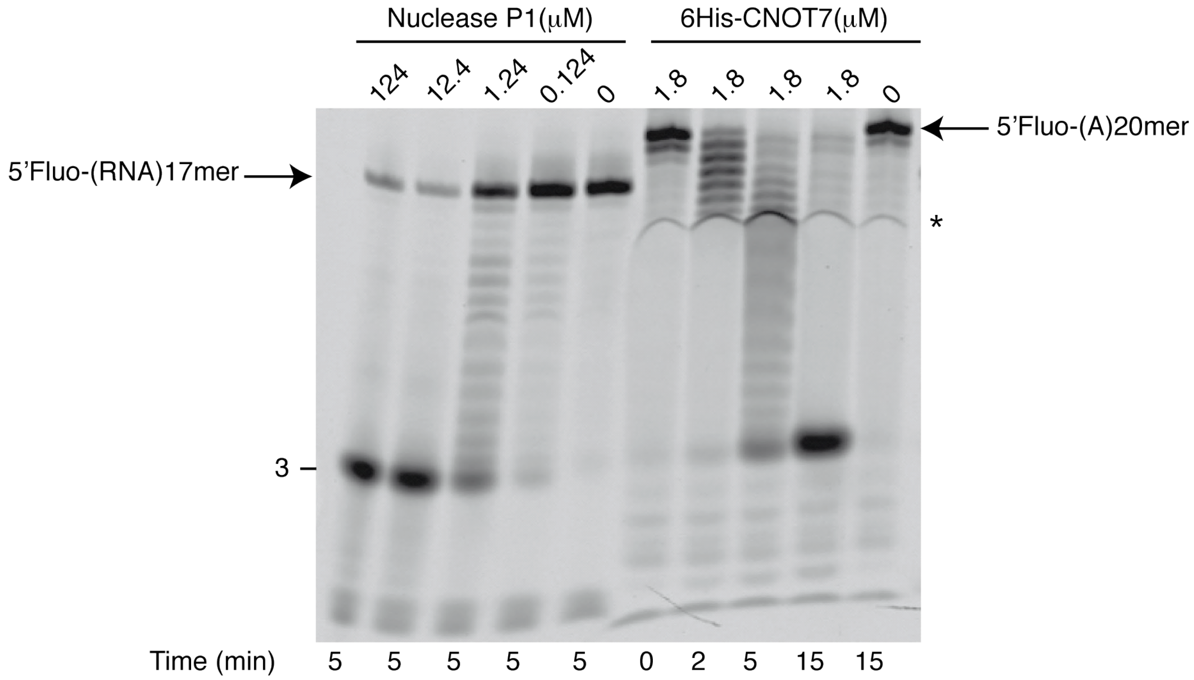

Supplementary Figure 5: The end product of the deadenylation reaction catalyzed by CAF1 (CNOT7 paralog) is less than 4 residues long.

A 5'-fluorescein-labeled synthetic RNA of 17 residues (100nM) was incubated 5 min at 50°C with decreasing concentrations of Nuclease P1 as indicated. In parallel, a 5'-fluorescein-labeled synthetic RNA of 20 A residues (100nM) was incubated with 6His-CNOT7 (1.8μM) during the indicated times. Both reactions were electrophoresed on a 15% denaturing polyacrylamide gel. An asterisk "*" indicates a distortion of migration due to the presence of HEPES in CAF1 deadenylase buffer. Note that on a 15% polyacrylamide gel, the end products of the reactions migrate as a single visible band whereas two bands appear with time on 8% polyacrylamide gels (see Fig. 3). This is due to the hydrophobic nature of the fluorescein tag that affects migration of oligonucleotides shorter than 4 residues.

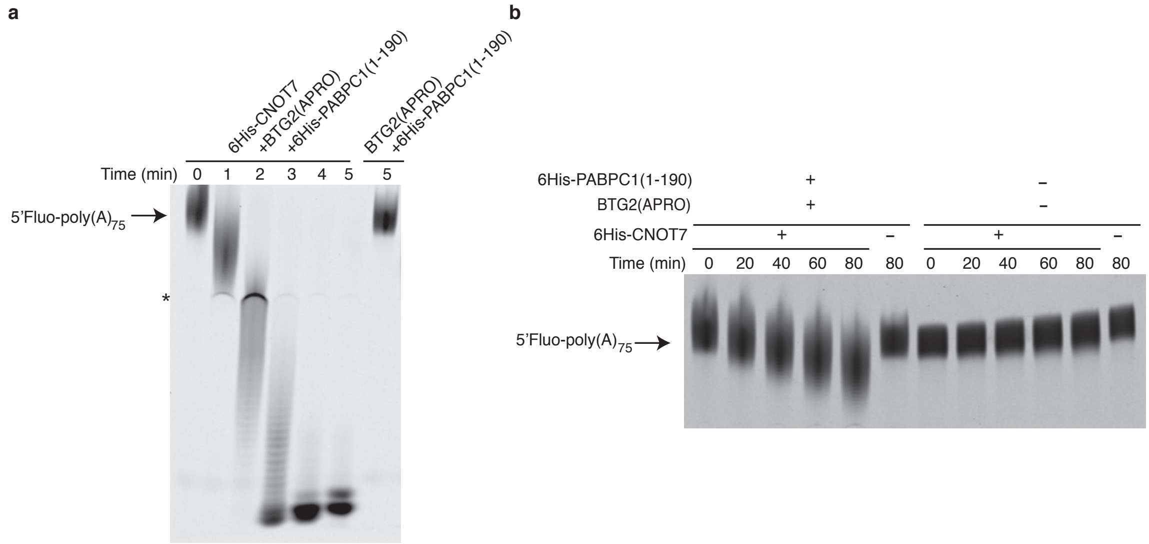

Supplementary Figure 6: Deadenylation by CAF1 (CNOT7 paralog) is progressive also in the presence of BTG2 APRO and PABPC1 RRM domains.

(**a**) – A 5'-fluorescein-labeled RNA of 75 A residues (100nM) was incubated at 30°C with purified BTG2(APRO) (1.8μM), 6His-PABPC1(1-190) (0.7μM) and with (or not) 6His-CNOT7 (1.8μM) for the times indicated, and electrophoresed on a 8% polyacrylamide gel. An asterisk "*" indicates a distortion of migration due to the presence of HEPES in CAF1 deadenylase buffer. Deadenylation rate is 18.9 nucleotides per minute in these conditions.

(**b**) – The 5'-fluorescein-labeled RNA of 75 A residues (100nM) was incubated and electrophoresed as in (a) but with reduced concentrations of BTG2(APRO) (50nM) and 6His-CNOT7 (50nM) and for the indicated times. The slightly altered migration of the substrate observed in the presence of 6His-PABPC1(1-190) is due to incomplete denaturation/removal of the PABPC1 protein and varies between experiments due to batches or reagents.

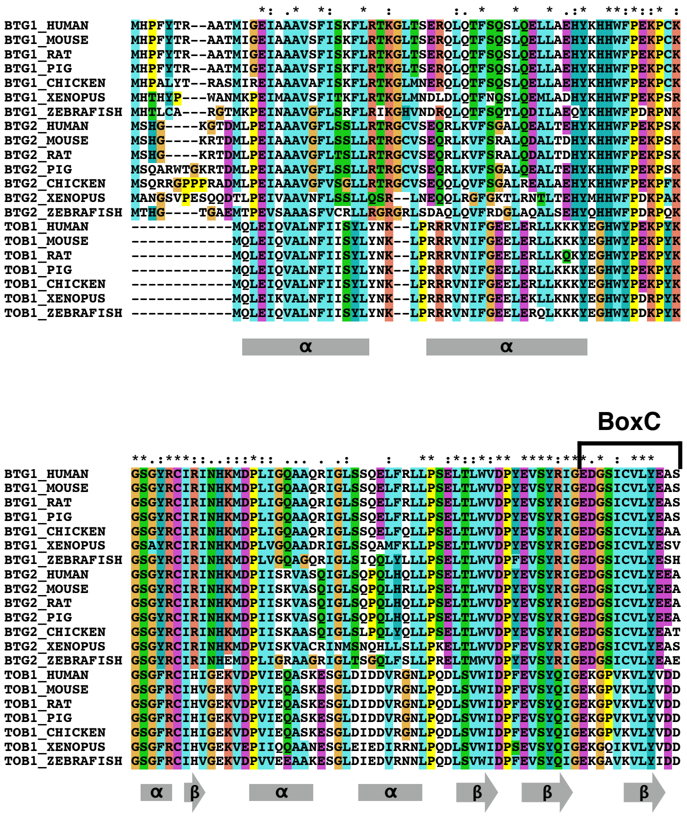

Supplementary Figure 7: Alignment of Apro domain region of BTG1, BTG2 and Tob1 proteins from various species.

The region shown starts at the initiator methionine and is truncated after the Apro domain. Coloring emphasizes amino acid properties conservation. The location of BoxC [1] is indicated. Alpha helices and beta-strands determined in the structure of Tob1 (PDB:2DR5) are depicted below the sequences. The following protein sequences were used: BTG1: human (Swissprot:P62324), mouse (Swissprot:P62325), rat (Swissprot:Q63073), pig (Trembl:A5YRP0), chicken (Swissprot:P34743), xenopus (Trembl:Q9PVQ0), zebrafish (Trembl:Q6PH73)), BTG2: human (Swissprot:P78543), mouse (Swissprot:Q04211), rat (Swissprot:P27049), pig (Trembl:B0F7G7), chicken (Trembl:R4GJS1), xenopus (Trembl:Q640A4), zebrafish (Trembl:Q9IB77)), and Tob1: human (Swissprot:P50616), mouse (Swissprot:Q61471), rat (Swissprot:Q8R5K6), pig (Trembl:B2BE68), chicken (Trembl:Q90VW0), xenopus (Trembl:Q28I92), zebrafish (Trembl:Q6QR52).

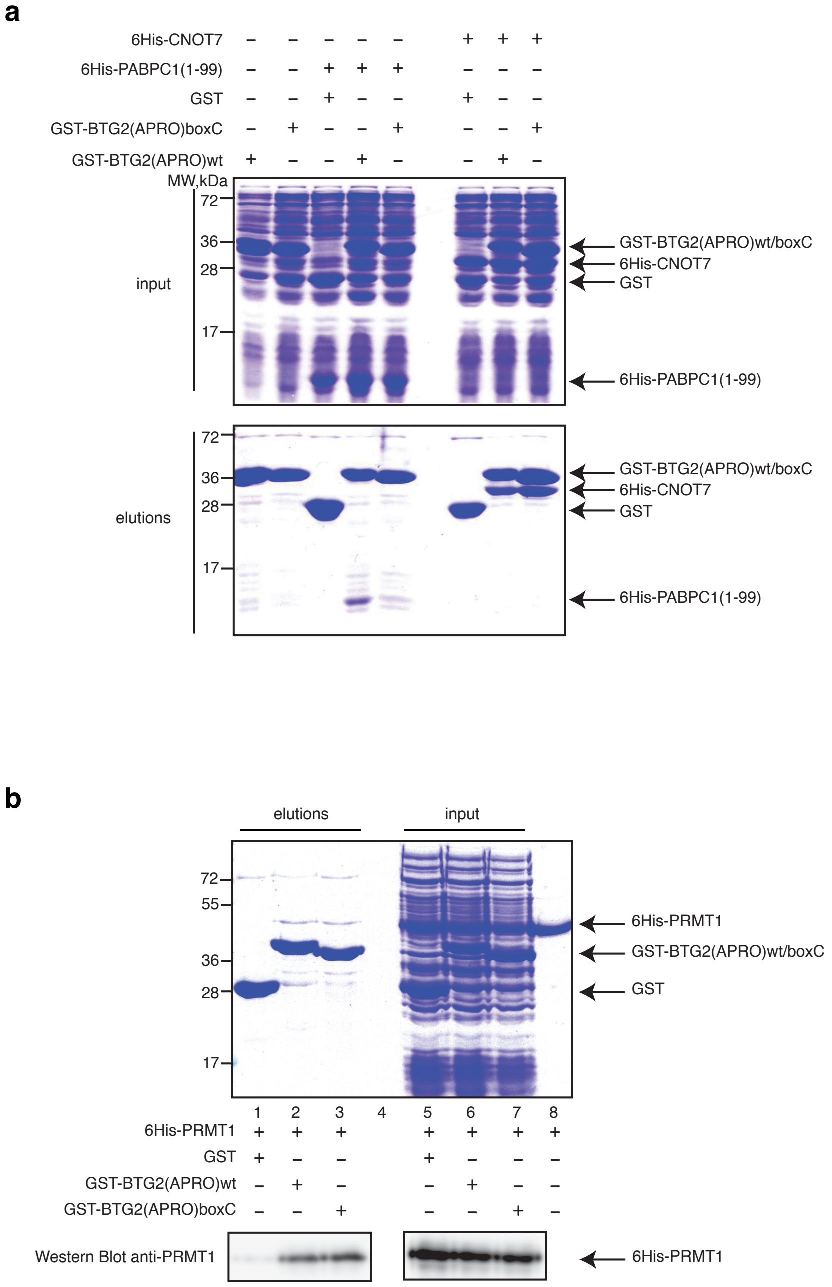

Supplementary Figure 8: Mutation of BTG2 boxC affects interaction with PABPC1 first RRM but not with CAF1 (CNOT7 paralog) or PRMT1.

(a) – 6His-PABPC1(1-99) or 6His-CNOT7 were co-expressed in bacteria with GST, GST-BTG2(APRO)wt or GST-BTG2(APRO)boxC as indicated. GST-tagged proteins were

purified on glutathione Sepharose resin and fractionated with co-purifying factors on 15% SDS-PAGE gel stained with Coomassie blue.

(**b**) – 6His-PRMT1 was purified to homogeneity (lane 8) and added to bacterial lysates expressing GST, GST-BTG2(APRO)wt or GST-BTG2(APRO)boxC as indicated. After an incubation of 15 min at 4°C, GST-tagged proteins were purified on glutathione Sepharose resin and fractionated with co-purifying factors on 12% SDS-PAGE gel stained with Coomassie blue. As the bands corresponding to 6His-PRMT1 were faint in the eluted materials, a Western blot analysis with anti-PRMT1 antibody (Sigma, clone PRMT1-171, # P1620; dilution 1 to 1000) was performed with diluted samples to ensure the identity of the protein.

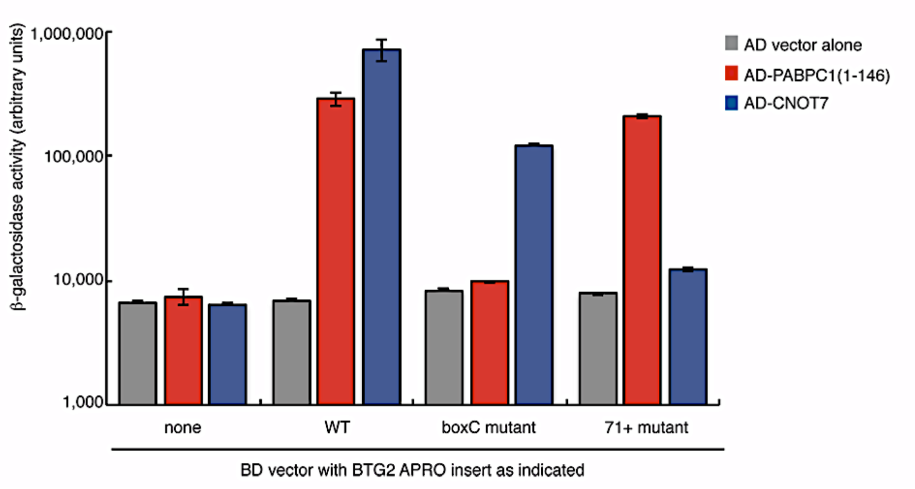

Supplementary Figure 9: Interaction of wild-type (WT) and mutant BTG2 APRO domains with the first RRM domains of PABPC1 and CAF1 (CNOT7 paralog) in yeast 2-hybrid assay.

71+ mutant: transposon-based insertion of 5 amino acids (HAAAN) after amino acid N71. Results for the boxC mutant (see Fig 4a) are shown for comparison. Interaction of wild-type and mutant BTG2 APRO domains with PABPC1 RRMs (AD-PABPC1(1-146)) and CAF1 (AD-CNOT7) was monitored by β-galactosidase assays. Activities are expressed in arbitrary units. Error bars (s.d.) correspond to two biological replicates.

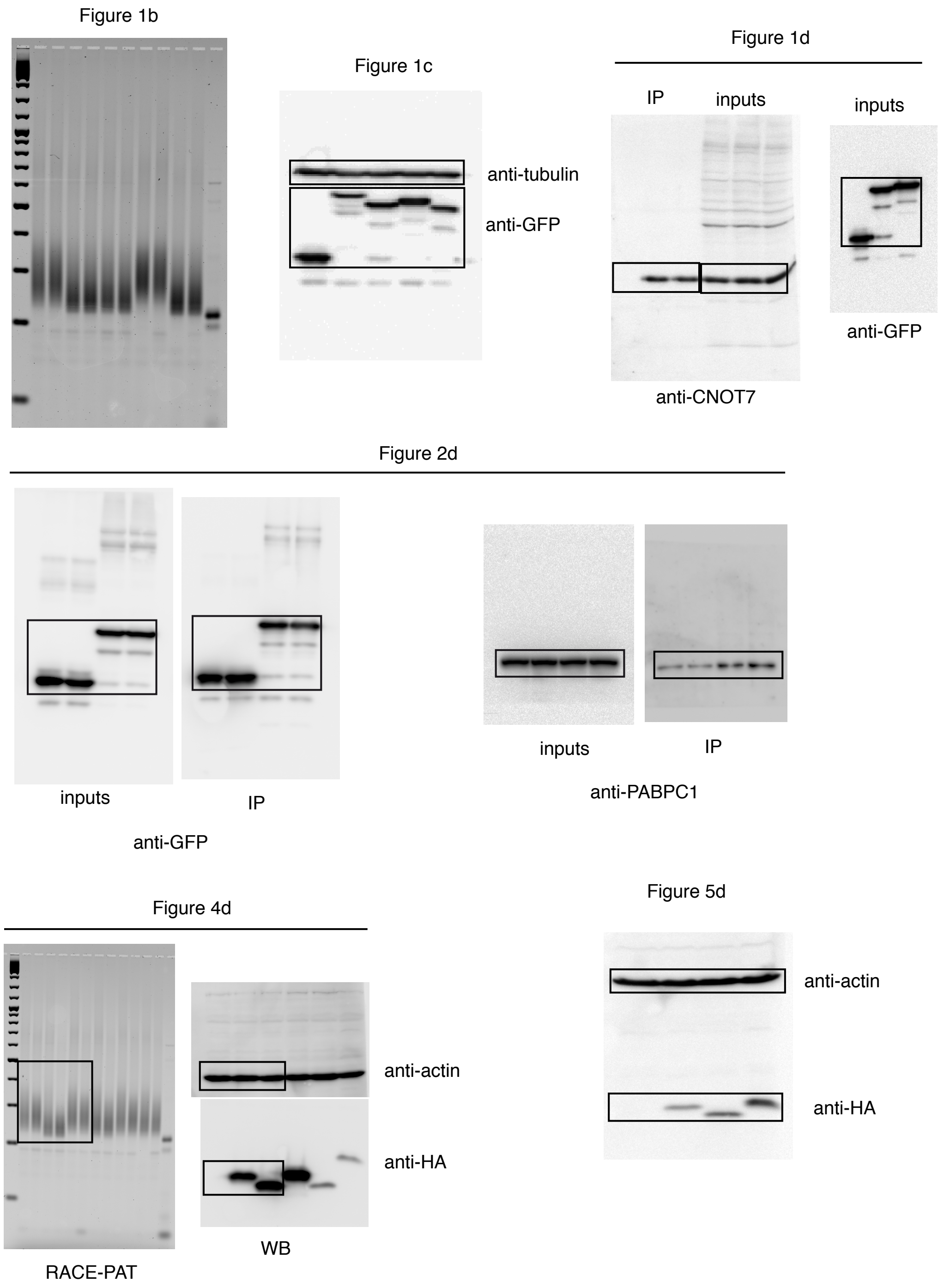

Supplementary Figure 10: Full gels and blots presented in the main text.

Darkness and/or contrast were modified to reveal blot dimensions.

SUPPLEMENTARY METHODS

Transcriptional pulse-chase experiments

HEK293 Tet-Off cells were transfected with 0.8 μg of the pTet-β-globin plasmid (pBS2800) and 1.6 μg of the BTG2 expressing plasmids in 6-cm diameter culture dishes. Immediately after transfection, doxycyclin (1ng/ml) was added in the medium of the cells to block transcription of the reporter. Two days after transfection, cells were washed and a 3-hour transcriptional pulse was performed before re-addition of doxycyclin (2 μg/ml). Chase times correspond to hours after doxycyclin addition and to times of RNA extraction.

Northern blot analysis

10 μg of total RNA was electrophoresed onto 1.4% agarose/6% formaldehyde gels and transferred to Hybond-N+ membranes (GE Healthcare). For poly(A)- controls, 10 μg of total RNA sample was digested with RNase H (Invitrogen) and oligo(dT) as recommended by the supplier. After phenol extraction and ethanol precipitation, 1:5 of the digestion was loaded on agarose gels. After transfer, blots were stained with methylene blue to check for equal loading and hybridized to probes synthesized by in vitro transcription with the T7 RNA polymerase (Promega). Hybridization signals were visualized with Typhoon 8600 (GE Healthcare) and quantified as followed: 15 identical adjacent rectangles encompassing the mRNA migration region from fully adenylated to deadenylated mRNAs were drawn to quantify the signal intensities that were plotted as a function of the distance of migration (indicative of poly(A) tail length).

Analytical ultracentrifugation

Analytical ultracentrifugation sedimentation velocity experiments were done at 4°C in a Beckman-Coulter ProteomeLab XL-I analytical ultracentrifuge at 50,000 rpm in a AN-50 Ti rotor with absorbance and interference detections. Sedimentation data were collected in 7 min intervals for formation of complexes between BTG2(APRO) and 6His-PABPC1(1-190) and in 5 min intervals for formation of complexes between [BTG2(APRO);6His-CNOT7] and 6His-PABPC1(1-190). The fitting of data was performed using SEDFIT software, version 14.1 and continuous sedimentation coefficient distribution model. The distributions obtained for each protein sample were integrated to determine the weight-average sedimentation coefficients as a function of protein concentrations and to generate Sw isotherms. The Sw isotherms were loaded into SEDPHAT for fitting with the hetero-association model A+B <-> AB to obtain an estimate of the Kd. Buffer density, buffer viscosity and protein partial specific volumes were calculated using SEDNTERP software. The software GUSSI was used to plot and integrate the sedimentation coefficient distributions.

Electrophoretic Mobility Shift Assays

1 picomole of the fluorescently labeled poly(A) substrate was incubated 10min at 30°C with increasing concentrations of proteins in the same buffer as for *in vitro* deadenylation assays in a final volume of 10µl. 2.5µl of loading buffer (50% glycerol, 0.05% bromophenol blue, TBE 1X) was added and the samples were electrophoresed at 4°C on native 6% polyacrylamide gels (ProtoGel, national diagnostics) that were visualized with Typhoon 8600 (GE Healthcare).

Purification of 6His-PRMT1

6His-PRMT1 was produced in E. coli BL21-CodonPlus strain (Stratagene) grown in auto-induction media (Formedium). Bacteria were lysed in buffer (20 mM Tris.HCl pH 8.0, 250

mM NaCl, 5% glycerol, 10 mM imidazole, 0.1 mM EDTA, 10 mM β-mercaptoethanol) supplemented with protease inhibitors (Complete Protease Inhibitor Cocktail EDTA-free, Roche) and Benzonase (BaseMuncher, Expedeon) by sonication. 6His-PRMT1 was purified by affinity chromatography on HisTrap FF crude column (GE Healthcare Life Sciences) followed by a size exclusion chromatography step on Superdex S200 10/300 column (GE Healthcare Life Sciences). Proteins were stored in gel filtration buffer containing 20 mM Tris.HCl pH 8.0, 250 mM NaCl, 5% glycerol, 10 mM imidazole, 0.1 mM EDTA, 10 mM β-mercaptoethanol.

SUPPLEMENTARY REFERENCES

1. Berthet, C. et al. Interaction of PRMT1 with BTG/TOB proteins in cell signalling: molecular analysis and functional aspects. *Genes Cells* **7**, 29-39 (2002).
